# Zinc anode-compatible in-situ solid electrolyte interphase via cation solvation modulation

**DOI:** 10.1038/s41467-019-13436-3

**Published:** 2019-11-26

**Authors:** Huayu Qiu, Xiaofan Du, Jingwen Zhao, Yantao Wang, Jiangwei Ju, Zheng Chen, Zhenglin Hu, Dongpeng Yan, Xinhong Zhou, Guanglei Cui

**Affiliations:** 1 Qingdao Industrial Energy Storage Research Institute, Qingdao Institute of Bioenergy and Bioprocess Technology, Chinese Academy of Sciences, Qingdao, 266101 P. R. China; 2 College of Chemistry and Molecular Engineering, Qingdao University of Science and Technology, Qingdao, 266042 P. R. China; 3 College of Chemistry, Beijing Normal University, Beijing Key Laboratory of Energy Conversion and Storage Materials, Beijing, 100875 P. R. China

**Keywords:** Batteries, Energy, Batteries

## Abstract

The surface chemistry of solid electrolyte interphase is one of the critical factors that govern the cycling life of rechargeable batteries. However, this chemistry is less explored for zinc anodes, owing to their relatively high redox potential and limited choices in electrolyte. Here, we report the observation of a zinc fluoride-rich organic/inorganic hybrid solid electrolyte interphase on zinc anode, based on an acetamide-Zn(TFSI)_2_ eutectic electrolyte. A combination of experimental and modeling investigations reveals that the presence of anion-complexing zinc species with markedly lowered decomposition energies contributes to the in situ formation of an interphase. The as-protected anode enables reversible (~100% Coulombic efficiency) and dendrite-free zinc plating/stripping even at high areal capacities (>2.5 mAh cm^‒2^), endowed by the fast ion migration coupled with high mechanical strength of the protective interphase. With this interphasial design the assembled zinc batteries exhibit excellent cycling stability with negligible capacity loss at both low and high rates.

## Abstract (web summary)

Zinc chemistry is not favourable to the formation of a solid electrolyte interphase as a result of its high redox potential. In a break with the traditional wisdom, the present authors realise ZnF_2_-rich hybrid SEI on Zn anode via the modulation of cationic speciation in a eutectic electrolyte.

## Introduction

Multivalent-ion (Mg^2+^, Zn^2+^, Ca^2+^, etc.) batteries (MIBs) are highly desirable for large-scale stationary energy-storage systems because of their abundant reservoir, environmental friendliness, intrinsic safety and comparable or even superior capacities to those of Li-ion counterparts^1–5^. Among the anode materials developed for MIBs, metallic Zn offers a better insensitivity in oxygen and humid atmosphere^6,7^, which broadens the availability of electrolytes and lowers the handling and processing costs. Additional enthusiasm for the Zn chemistry is stimulated by its high volumetric capacity (5855 Ah L^−1^), superior to Li (2061 Ah L^−1^), Ca (2072 Ah L^−1^), and Mg (3833 Ah L^−1^) counterparts^8^. Indeed, since the “rediscovery” of the rechargeable Zn-ion batteries (ZIBs), new cathode materials and Zn-storage mechanisms have enjoyed substantial achievements in the last few years^8,9^. However, there is a fly in the ointment: the suboptimal cycling efficiency resulting from uncontrolled dendrites and notorious side-reactions occurred at the Zn-electrolyte interface (especially for aqueous electrolytes) restricts the development of real rechargeable ZIBs and their broad applicability^6,10,11^.

Actually, intensive previous investigations have been dedicated to handling these Zn-related issues, such as introducing additives into electrolytes or electrodes, constructing nanoscale interface and designing hierarchical structures^12–15^, but still suffer from a low Coulombic efficiency (CE). Very recently, highly concentrated electrolytes were introduced to stabilize the Zn anode by regulating the solvation sheath of the divalent cation^16–18^, which is a feasible approach to reducing water-induced side-reactions and improving Zn plating/stripping CE. Unfortunately, the underlying mechanism on the inhibition of Zn dendrites has not been entirely understood yet. Even today, the Zn-electrolyte interface instability remains challenging, and a broadly applicable interfacial protection strategy is highly desired yet largely unexplored, especially compared with the rapid progress regarding the effective utilization for alkaline metal (Li or Na, etc.) anodes.

When encountered with a similar dilemma of intrinsic limitations on anodes, Li-ion batteries (LIBs) offer a tactful response: in situ formation of a solid electrolyte interphase (SEI). Admittedly, this SEI is highly permeable for Li ions and prevents excess Li consumption by blocking solvents and electrons^19,20^. Importantly, via electrolyte modulation (e.g., introduction of F-rich species), additional unusual functionalities can also be achieved, in particular concerning the dendrite suppression and long-term cycling for Li-metal batteries at high CE or high rate^20,21^. However, such SEI response has always been associated with aprotic electrolytes. Given the much higher redox potential of Zn/Zn^2+^ couple (−0.76 V vs. NHE) compared with that of Li/Li^+^ (−3.04 V vs. NHE), routine anions and organic solvents are difficult to decompose reductively before Zn deposition. Despite the great appeal for aqueous Zn anodes, the competitive H_2_ evolution reaction inevitably occurred during each recharging cycle makes this in situ protection mechanism infeasible^22^, while the local pH change induces the formation of ionically insulating byproducts which has been also faced in other multivalent metal anodes^23,24^. Hence, another possibility for designing reliable Zn-anode SEI is to look beyond the conventional water-based and organic-solvent electrolytes.

We explore the in situ formation of a ZnF_2_-rich, ionically permeable SEI layer to stabilize Zn electrochemistry, by manipulating the electrolyte decomposition based on a eutectic liquid with peculiar complexing ionic speciation. Regulating the solvation structure (either locally or totally) has been verified and found to be an effective strategy for shifting the reductive potentials of electrolyte components^20,21,25^; however, due to the high charge density of Zn^2+^, Zn salts do not readily dissociate in common solvents over a wide concentration range, resulting in limited control over the coordination properties. As a new class of versatile fluid materials, the deep eutectic solvents (DESs), generally created from eutectic mixtures of Lewis or Brønsted acids and bases that can associate with each other, have been found to be interesting on account of their excellent dissolution ability, even for the multivalent metal salts and oxides^26,27^. Remarkably, characterized by highly adjustable compositions and rich intermolecular forces, DESs are also expected to accommodate concentrated ionic species and have aided the development of alternative media for electrochemistry^27,28^.

Here in this work, based on a new DES composed of acetamide (Ace) and Zn(TFSI)_2_, a large portion of TFSI^−^ is found to coordinate to Zn^2+^ directly in the form of anion-containing Zn complexes ([ZnTFSI_*m*_(Ace)_*n*_]^(2−*m*)+^, *m* = 1–2, *n* *=* 1–3), which induces the preferential reductive decomposition of TFSI^−^ prior to Zn deposition. Correspondingly, a well-defined anion-derived SEI layer compositionally featured with a rich content of mechanically rigid ZnF_2_ and Zn^2+^-permeable organic (S and N) components can be obtained during the initial cycling. This SEI-coated Zn anode is stabilized to sustain long-term cycling (>2000 cycles; average Zn plating/stripping CE of 99.7%), and enables a highly uniform Zn deposition even at a high areal capacity of 5 mAh cm^−2^, without short-circuit or surface passivation. Moreover, the transformed interfacial chemistry has been further confirmed by the unprecedented reversibility of Zn redox reactions upon implanting the SEI-coated Zn anodes into cells with routine aqueous electrolytes. With this in situ anode protection, ZIBs paired with a V_2_O_5_ cathode accomplish the cyclability of 92.8% capacity retention over 800 cycles (99.9% CEs after activation), and are demonstrated to cycle up to 600 times along with a capacity fading of only 0.0035% cycle^−1^ under a practical cathode-anode coupling configuration (Zn:V_2_O_5_ mass ratio of 1:1; areal capacity of >0.7 mAh cm^−2^). As per our knowledge, it is the first successful attempt to in situ construct reliable SEI on Zn anode, providing fresh insights for all multivalent chemistries confronted with the same requirements at anodes.

## Results

### The new Zn(TFSI)_2_/Ace eutectic solution and physicochemical properties

Recent progress on new electrolytes has demonstrated that better control over the metal coordination environment provides more possibilities for achieving unique properties beyond routine views, such as significantly extended electrochemical window, enhanced oxidative/reductive stability and unusual ion-transport behavior^29^. In fact, there have been reports on ZnCl_2_-based DESs, in which various complex anionic (e.g., [ZnCl_3_]^−^, [ZnCl_4_]^2−^, and [Zn_3_Cl_7_]^−^) and cationic (e.g., [ZnCl(HBD)_*n*_]^+^; HBD, hydrogen bond donor) Zn^2+^ species can be detected^30,31^. The Cl-containing solutions, however, are corrosive to common battery components especially at high operation voltages, and are not readily available in forming a stable and ionically conducting interphase for Zn anodes. As an alternative anion to form DESs, TFSI^−^ is generally considered to be a decomposition source to promote the formation of uniform SEI and thus has aroused our concern^12,32^. Besides, the binding energy of TFSI^−^ to metal ions appears to be relatively lower compared with those of other conventional anions such as BF_4_^−^, PF_6_^−^, allowing the TFSI-based salts to dissociate easily^33,34^. Based on the above considerations, the Zn(TFSI)_2_-based eutectic solvent (ZES) with Ace as the HBD was selected as a promising Zn electrolyte.

As shown in Supplementary Fig. 1, the ZESs are homogenous and transparent liquids at ambient-temperature when Zn(TFSI)_2_ and Ace were blended in a predetermined molar ratio range (1:9−1:4). Correspondingly, they are denoted as ZES 1:*x* solutions (*x* = 4, 5, 7, and 9). The lowest-eutectic temperature of ZES is found to be −51.51 °C at a molar ratio of 1:9, and rises to −35.70 °C with the molar ratio increased to 1:4 (Supplementary Fig. 2 and Supplementary Table 2). Furthermore, no phase change is observed in all ratios blow 100 °C, and weight losses are only about 4.3% (1:9) and 3.3% (1:4) after heating at 100 °C (Supplementary Figs. 2, 3), reflecting the thermal adaptability of ZESs in the operating temperature region. This wide temperature range of liquid state is in contrast to the highly concentrated electrolytes that suffer from salt precipitation at low temperatures^35^; simultaneously, the cost issue of the salt-concentrated method can be alleviated^29^.

### Solution structure analysis of ZES

**Fig. 1:**
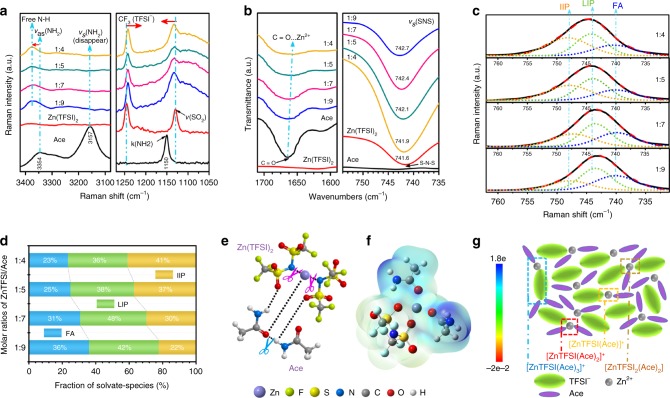
Structure analysis of ZESs and identity of the ionic species. **a** Raman, **b** FTIR, and **c** Fitted Raman spectra of ZESs with different Zn(TFSI)_2_/Ace molar ratios (1:9–1:4). Solid and dashed lines denote experimental spectra and fitting curves, respectively. **d** Solvate species distribution in ZESs (free anions (FA), loose ion pairs (LIP) and intimate ion pairs (IIP)), all obtained from the fitted Raman spectra. **e** Schematic diagram of the interplay among Zn^2+^, TFSI^−^, and Ace to form eutectic solutions. **f** Molecular electrostatic potential energy surface of [ZnTFSI(Ace)_2_]^+^ (*C*_2_-O-П, bidentate coordination of TFSI^−^) based on density functional theory (DFT) simulation. Electron density from total self-consistent-field (SCF) density (isoval = 0.001). **g** Illustration of representative environment of active Zn species within the ZES.

The formation mechanism of the ZES was explored by various spectrum analyses. The Raman bands at 3354 and 3157 cm^−1^ in solid Ace correspond to the asymmetric and symmetric NH stretching, respectively (Fig. 1a, left). Upon the introduction of Zn(TFSI)_2_, the 3354 cm^−1^ band moves to 3380 cm^−1^ while the 3157 cm^−1^ band disappears, suggesting the breaking of H-bonding between Ace molecules. The SO_3_ and CF_3_ groups of TFSI^−^ are proved to be sensitive to the cation–anion and anion–solvent interactions^36,37^. Once eutectic liquid formed, there is a strong interaction between the NH_2_ group on Ace and the SO_2_ group on Zn(TFSI)_2_, as implied by the overlap of the bands at 1150 cm^−1^ and 1128 cm^−1^ (Fig. 1a, right)^38,39^. From fourier transform infrared spectroscopy (FTIR) spectra of ZESs (the left panel of Fig. 1b), an evident change appears at the C=O stretching region of Ace, where the 1665 cm^−1^ band red-shifts slightly to 1654 cm^−1^ accompanied by obvious broadening in comparison to the pristine Ace. This is in line with the formation of metal-oxygen coordination between Zn^2+^ and C=O group (Fig. 1e)^39–41^. These intermolecular interactions between components jointly weaken the respective bonds of pristine components, resulting in eutectic solutions (for more details, see Supplementary Figs. 4, 5).

For the highly concentrated electrolytes proposed for LIBs, organic anions tend to coordinate to Li^+^ and exist dominantly in associated states (i.e., contact ion pairs or ionic aggregates), which effectively tunes the molecular frontier orbit properties of the electrolyte solutions^42^. In theory, this strategy can also be anticipated in multivalent metal electrochemistries, but no one has yet achieved it partially due to shortage of reliable electrolyte systems at present. Inspirationally, the associated Zn^2+^-TFSI^−^ states have been found by reasonably introducing a neutral ligand (i.e., Ace) to create anion-containing Zn^2+^ species in this work, which is verified in detail below Fig. 1b (right panel) compares the shift of the *υ*_s_ (SNS) peak (TFSI^−^)^43,44^ as the salt concentration increases, with crystalline Zn(TFSI)_2_ as the reference (bottom trace). Apparently, this vibration mode is rather susceptible to the change of the TFSI^−^ environment^42^, slightly drifting from 742.7 cm^−1^ at 1:9 to 741.9 cm^−1^ at 1:4. Essentially, the latter is identical to that in crystal lattice (741.6 cm^−1^), indicative of a possible pronounced interionic attraction in ZESs^43^. Turning to the Raman vibration mode of TFSI^−^ at the same region (Fig. 1c), a deconvolution analysis shows that the Raman band consists of three modes at 740, 744, and 748/747 cm^−1^, arising from free anions (FA)-(#Zn^2+^ = 0), loose ion pairs (LIP)-(#Zn^2+^ = 1), and intimate ion pairs (IIP)-(#Zn^2+^ = 1), respectively^22,37,45^. In all cases of the ZES system, albeit without obvious ionic aggregates (AGG; the anions are coordinated to two or more cations)^37^, the ubiquitous presence of cation–anion coordination can be identified. In 1:7 and 1:9 solutions, the majority of TFSI^−^ anions exist as long-lived LIPs, suggesting the dominant monomeric Zn species coordinated by TFSI^−^, while the ionic association becomes stronger with more IIPs formed at relatively higher salt contents (1:4 and 1:5 solutions) (Fig. 1d). Effects related to salt concentration are also imposed on drastic variation in viscosity and ion conductivity (Supplementary Fig. 13c).

The high-resolution mass spectra (HRMS) of ZESs testify the existence of LIPs and IIPs in all given ratios. Typically, distinct signals of various cationic TFSI^−^-containing complexes ([ZnTFSI(Ace)]^+^ at *m*/*z* = 403, [ZnTFSI(Ace)_2_]^+^ at *m*/*z* = 462, and [ZnTFSI(Ace)_3_]^+^ at *m*/*z* = 521) can be detected, but without evidence of free Zn^2+^ ions (Supplementary Fig. 6). Moreover, the variation trend of these cationic peak intensities qualitatively indicates a more pronounced ionic association upon increasing the Zn-salt content, in line with the above Raman results (Fig. 1c, d). It should be noted that the only anionic species of TFSI^−^ found in HRMS suggests a low possibility of anionic Zn complexes (monomeric) with more than two associated TFSI^−^ anions (Supplementary Fig. 7).

Theoretical simulations were performed to further identify the ion speciation of ZESs. In both cases of mixtures (1:7 and 1:4), molecular dynamics (MD) simulations predict a competition between the Ace and TFSI^−^ for coordination to Zn^2+^ cations (Supplementary Fig. 8). For the 1:7 ratio, one TFSI^−^ anion (on average) could be observed in each Zn^2+^ primary solvation sheath, typically in the form of the [ZnTFSI(Ace)_2_]^+^ solvate (Supplementary Fig. 8a, b). However, in 1:4 ZES, where only four Ace molecules per Zn(TFSI)_2_ are involved in eutectic solution formation, a lower Ace population is available for Zn^2+^ solvation and H-bonding with TFSI^−^ anions simultaneously^42^; instead, more TFSI^−^ anions enter the Zn^2+^ solvation sheath (Supplementary Fig. 8c, d). The fraction of neutral Zn complexes coordinated by two TFSI^−^ anions is thus expected to increase, whereas no three TFSI^−^ coordination case was found (Supplementary Fig. 8d). Apparently, ZES is a system featured with the existence of anion-associated Zn solvates, and the ionic interplay strength can be tuned through simple regulation of the Zn(TFSI)_2_/Ace ratio. By virtue of structural flexibility, the TFSI^−^ anion may be coordinated in varying ways to Zn^2+^ cations^37^, incurring dynamic equilibria of cationic or neutral species with various configurations (Fig. 1g).

Given the fact that TFSI^−^ is more likely to form bidentate coordination to a single cation than other common anions (i.e., PF_4_^−^, ClO_4_^−^, and BF_4_^−^)^46^, the local atomic configurations of Zn complexes were investigated theoretically. The density functional theory (DFT) geometry optimization of [ZnTFSI(Ace)_*n*_]^+^ complexes verifies the preference of the C=O group of Ace and both two O atoms of TFSI^−^ for the coordination with the central Zn^2+^ cation (Supplementary Figs. 9, 11). The [ZnTFSI(Ace)_2_]^+^ structure with bidentate coordination by TFSI^−^ possesses the most uniform molecular electrostatic potential energy surface distribution along with relatively low total binding energy (Fig. 1f and Supplementary Figs. 9, 11, 12), in reasonable agreement with the predominant signal of cationic species observed from HRMS. Note that the steric-hindrance effect caused by the bulky TFSI^−^ also dictates the identity of solution species. This can be reflected by the absence of anionic Zn solvates and lower tendency of bidentate coordination in [ZnTFSI_2_(Ace)_*n*_] complexes (Supplementary Fig. 10).

### Electrochemical and ion-transport properties of the ZES

On the optimization of electrolytes, the ZES with a molar ratio of 1:7 was found to possess a relatively high ionic conductivity (0.31 mS cm^−1^), a low viscosity (0.789 Pa·s) at 25 °C, as well as an optimum Zn/Zn^2+^ redox activity (Supplementary Fig. 13 and Supplementary Table 3). Taking the physical/chemical properties and cost factors into consideration, we chose the molar ratio of 1:7 as the main research object (for the selection of the control group see Supplementary Figs. 14, 15). Supplementary Fig. 16 displays the voltametric response of ZES as compared with an aqueous electrolyte of 1 M Zn(TFSI)_2_. It is evident that due to the water-splitting reaction, the potential window of 1 M Zn(TFSI)_2_ is restricted to 1.9 V (vs. Zn/Zn^2+^). In contrast, the ZES provides an expanded anodic stability limit of 2.4 V (vs. Zn/Zn^2+^), also outperforming those of DESs formed by other common Zn salts (e.g., Zn(ClO_4_)_2_, Zn(CH_3_COO)_2,_ and Zn(BF_4_)_2_) (Supplementary Fig. 17). The features regarding thermal and electrochemical stabilities allow ZES to be coupled with a wide range of high-voltage cathodes and to work at elevated temperatures, enabling elaborate optimizations of operating conditions in batteries.

In addition, the ZES exhibits a much higher Zn^2+^ transference number (0.572, Supplementary Fig. 18a) as compared with those of other available Zn liquid electrolytes (0.2–0.4)^47^. This effective migration of metal cations is most likely accounted for by the peculiar cationic Zn solvates with tethered anions^48^, and the resulting limited transport for negative charge carriers, which is analogous to the observations in highly concentrated electrolytes^29,49^. Furthermore, the high Zn^2+^ transference number also implies that the ion-transport manner in ZESs differs from those observed in the conventional dilute electrolytes; the active Zn^2+^ species might obey underlying hopping-type ion-transport mechanisms (Zn^2+^ ions move from one anion to another through Lewis basic sites on TFSI^−^ with the aid of Ace matrix)^29,49^. As shown in Supplementary Fig. 18b, the diffusion coefficient of the active Zn species through ZES is 1.66 × 10^−6^ cm^2^ s^−1^, exceeding ionic liquid-based electrolytes reported^50,51^. These remarkable kinetic properties make for powering high-rate devices^50,52,53^.

### High Zn/Zn^2+^ reversibility and uniform Zn deposits

**Fig. 2:**
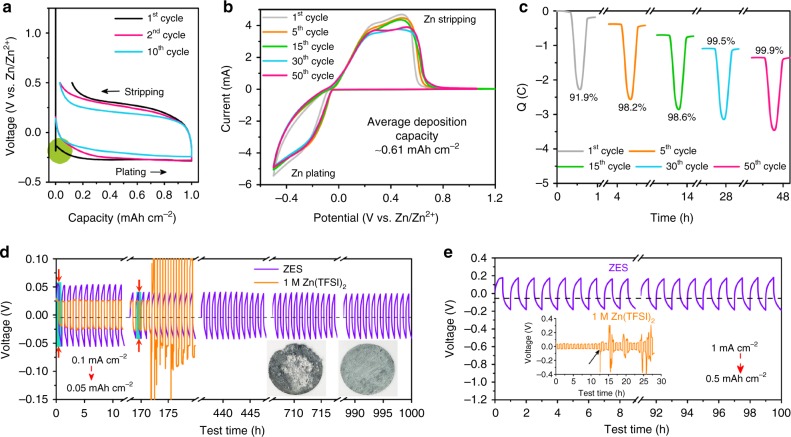
Zn plating/stripping behaviors in ZES. **a** Voltage profiles of galvanostatic Zn plating/stripping with the maximum oxidation potential of 0.5 V (vs. Zn/Zn^2+^) in ZES at a rate of 0.5 mA cm^−2^ (1.0 mAh cm^−2^). The working and counter electrodes are Ti and Zn, respectively. **b** Cyclic voltammetry (CV) curves of Zn plating/stripping in ZES at a scan rate of 1 mV s^−1^ with a potential range of −0.5–1.2 V and an average deposition capacity of ~0.61 mAh cm^−2^. The working and counter electrodes are Ti and Zn, respectively. **c** Chronocoulometry curves of Zn plating/stripping in ZES based on CV. Voltage responses of Zn/Zn symmetric cells **d** in ZES and 1 M Zn(TFSI)_2_ electrolytes at 0.1 mA cm^−2^ (0.05 mAh cm^−2^ for each half cycle) for 1000 cycles (insets: the optical images of the cycled Zn after 180 cycles in 1 M Zn(TFSI)_2_ (left) and 2000 cycles in ZES (right)), and **e** in ZES electrolyte (inset: in 1 M Zn(TFSI)_2_ electrolyte) at 1 mA cm^−2^ (0.5 mAh cm^−2^ for each half cycle).

The CE of Zn plating/stripping, the most critical parameter responsible for the redox reversibility, was first investigated in Zn/Ti cells with a galvanostatic capacity of 1 mAh cm^−2^ (0.5 mA cm^−2^) (Fig. 2a; Supplementary Fig. 19). Of note, the CE of the first 10 cycles in ZES rises gradually to above 98.0%; instead, the inferior CE of <70% was obtained in 1 M Zn(TFSI)_2_ (Supplementary Fig. 20a, b) under identical conditions, which could be ascribed to the severe parasitic reactions that simultaneously occurred during Zn deposition^17^, along with uncontrolled dendrites (Supplementary Fig. 20d)^12,54^. Interestingly, an overpotential of 0.185 V is required for the 1st cycle in ZES while roughly 0.1 V needed in the following cycles (Fig. 2a, green circle), which suggests the increase in surface area as well as the progressively improved stability induced by stepwise generation of the in situ formed interphase^55,56^. The relatively lower CE of ZES in initial cycles might originate from the consumption of active Zn^2+^ for such interfacial activation. Additional support for our hypothesis comes from the post-mortem scanning electron microscopy (SEM) observation. It is apparent that a protective coating layer formed on the Ti surface deposited by flat and dense Zn (Supplementary Fig. 20c), essentially differing from the tanglesome deposits in 1 M Zn(TFSI)_2_ (Supplementary Fig. 20d). As another reliable method^16,57^, cyclic voltammetry (CV) was further applied to evaluate CE of ZES at an average deposition capacity ~0.61 mAh cm^−2^ (Fig. 2b). Corresponding chronocoulometry curves (Fig. 2c) reveal that the plating/stripping is highly reversible; the CE approaches 100% after the initial 30 conditioning cycles (an average CE of 99.7% for 200 cycles; see Supplementary Fig. 21). From this aspect, compared with other reported Zn electrolytes (see Supplementary Table 4)^16,17,58^, ZES provides a more promising route for the realization of secondary Zn-metal cells to charge for hundreds of times, especially when the excess of Zn anode is limited.

The superior performance of ZES for supporting the Zn anode was also demonstrated under galvanostatic conditions in a Zn/Zn symmetric configuration. Despite of a slightly larger polarization, all cells using ZES exhibit more sustainable electrochemical cycling in contrast to those with 1 M Zn(TFSI)_2_. As viewed from Fig. 2d, the overpotential experienced a gradual decrease (from 55 to 39 mV) upon cycling at 0.1 mA cm^−2^ in ZES, conforming to the formation process of SEI. As the rate was raised to 0.5 mA cm^−2^, the same cell with ZES continued to operate steadily for another 1000 h (Supplementary Fig. 22a). Note that the surface morphology of cycled Zn in ZES is visually uniform (Fig. 2d inset right and Supplementary Fig. 22c), while characteristic Zn protrusions are shown in the case using 1 M Zn(TFSI)_2_ (Fig. 2d inset left and Supplementary Fig. 22b). Even cycled at an elevated rate of 1 mA cm^−2^ and a capacity of 0.5 mAh cm^−2^, the Zn/Zn cell with ZES also maintains an impressive stability without voltage fluctuation, which lays the foundation for designing high-rate ZIBs. In sharp contrast, an erratic voltage response with the rapidly rising overpotential occurred after only 15 cycles in 1 M Zn(TFSI)_2_ (Fig. 2e inset). Given that side-reactions at the electrolyte–electrode interface are considered to be more competitive at relatively low rates^12^, further interrogation of the ZES electrolyte was carried out at 0.01−0.05 mA cm^−2^ (charge/discharge interval being extended to 10 h, Supplementary Fig. 23). The Zn/Zn^2+^ redox reactions remain reversible and steady with a cycling life over 200 h.

The Zn-electrolyte interface stability has been detected by the sensitive electrochemical impedance spectroscopy (EIS). The charge-transfer resistance of symmetric Zn/Zn cell using 1 M Zn(TFSI)_2_ keeps increasing with cycling, reaching 356 ohm after 15 cycle (Supplementary Fig. 24). In the case of ZES, a much better interfacial compatibility can be obtained and the charge-transfer resistance maintains steady after 15th cycles. This significant difference is ascribed to, in part, the competing H_2_ evolution reaction (Supplementary Fig. 25a) and the accumulation of undesirable passivating byproducts (such as Zn(OH)_2_, *x*ZnCO_3_•*y*Zn(OH)_2_•*z*H_2_O and ZnO) on Zn anode surface upon cycling in 1 M Zn(TFSI)_2_ (Supplementary Fig. 25b). By contrast, the cycled Zn obtained from ZES presents well-defined X-ray diffraction (XRD) peaks, agreeing well with the Zn reference (PDF#99-0110). The time evolution of interface resistance under open circuit conditions further verifies the chemical stability of the metallic Zn in ZES (Supplementary Fig. 26).

**Fig. 3:**
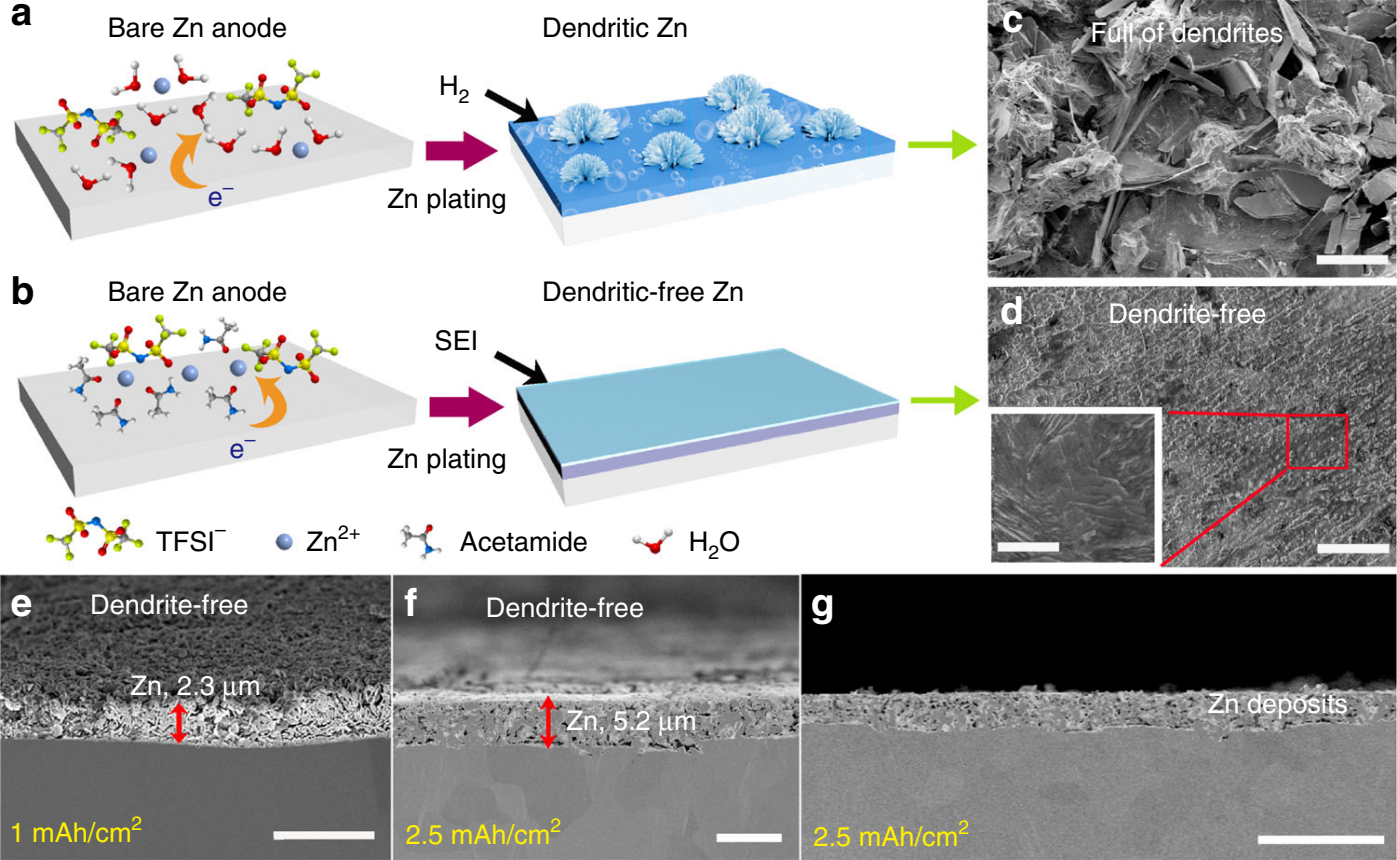
Effect of ZES and as-obtained SEI layer on Zn deposition. **a** Zn dendrite growth along with H_2_ evolution observed in 1 M Zn(TFSI)_2_ and **b** SEI-regulated uniform Zn deposition in ZES. SEM images of Zn deposits using **c** 1 M Zn(TFSI)_2_ and **d** ZES electrolytes at 1 mA cm^−2^ (0.5 mAh cm^−2^). **e**–**g** Cross-sectional SEM images of Zn deposits which were obtained in ZES electrolyte with 1 mAh cm^−2^ (0.25 mA cm^−2^) (**e**) and 2.5 mAh cm^−2^ (0.5 mA cm^−2^) (**f**) Zn on Zn substrate, respectively. **g** A lower-magnification image of panel (**f**) showing a large area of uniform deposition. Scale bar: 50 μm for (**c**), (**d**); 2 μm for (**e**); 5 μm for (**f**); 20 μm for (**g**) and inset in (**d**).

The utilization of ZESs along with the possible in situ SEI layer can have a large impact on the Zn deposition. Upon deposition capacity of 0.5 mAh cm^−2^, loose structures with uncontrolled dendritic Zn growth appeared in 1 M Zn(TFSI)_2_ (Fig. 3a, c), which surely accounts for the low CE (Supplementary Fig. 20a). This is not the case for our ZES electrolyte, as SEM images in Fig. 3d, e clearly show dendrite-free and smooth Zn deposits, even at higher capacity of 2.5 mAh cm^−2^. Based on the cross-sectional views (Fig. 3f, g), thickness of the deposited Zn layer is about 5.2 μm, in line with the expected value 4.3 μm for capacity of 2.5 mAh cm^−2^, which represents the dense Zn coating by electrodeposition in ZES. Notably, with increasing current densities (Fig. 3e, f), the Zn deposits become more compact (theoretical/actual thickness: 1.7/2.3 at 0.5 mA cm^−2^ less than 4.3/5.2 at 0.25 mA cm^−2^) while the particle size decreases slightly, following the classical nucleation theory^56^. It is also visible that the Zn anode after deposition is covered by a thin surface layer (Fig. 3d inset), also corresponding to the surface modification. Thus, it is reasonable to assume that this additional Zn-electrolyte interphase dictates the reversible Zn/Zn^2+^ redox with efficient Zn^2+^ transport and deposition (Fig. 3b).

### Formation mechanism and chemical composition analysis of the SEI layer on Zn anode

**Fig. 4:**
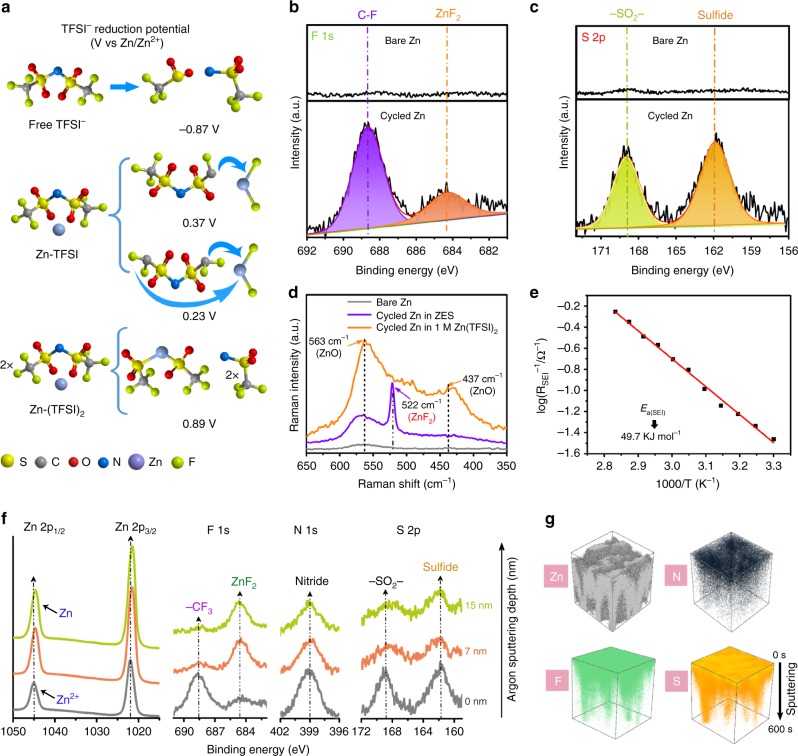
Experimental and theoretical investigations on the existence and the composition of the Zn-compatible SEI layer. **a** Predicted reduction potentials by DFT calculations. XPS spectral regions for **b** F 1s and **c** S 2p of the surface of bare Zn (top) and after cycled Zn (bottom), respectively. **d** Raman spectra of the cycled Zn anode in ZES and 1 M Zn(TFSI)_2_. **e** Arrhenius behavior of the reciprocal resistances corresponding to interfacial components and the activation energy derived for the in situ formed SEI. **f** XPS spectral regions for Zn 2p, F 1s, N 1s, and S 2p at various argon (Ar^+^) sputtering depths on the SEI accumulated on Zn substrate. **g** Three-dimensional view of Zn, N, F, and S elements distributions of SEI in the time-of-flight secondary-ion mass spectrometry (TOF-SIMS) sputtered volumes. The SEI-coated Zn anode was obtained after 20 cycles of galvanostatic plating/stripping in ZES electrolyte (Zn/Zn cells at 0.5 mA cm^−2^ with a capacity of 1 mAh cm^−2^ for each half cycle).

A uniform SEI can be rationally constructed by introducing selected elements and/or compounds (such as F-donating salts and solvents) that decompose beneficially on alkaline metal anodes^59^, but due to the higher redox potential of the Zn/Zn^2+^ couple (−0.76 V vs. NHE) than that of free TFSI^−^ (−0.87 V vs. Zn/Zn^2+^)^42^, the reductive TFSI^−^ decomposition can hardly take place before Zn deposition. In our case, by virtue of the intrinsic ion-association network in present eutectic liquid, a marked change of the TFSI^−^ coordination environment has been confirmed (Fig. 1), making the anion-derived SEI formation for metallic Zn possible. DFT calculations demonstrate the altered reduction potential of TFSI^−^ by its intimate interaction with Zn^2+^ (Fig. 4a). The Zn^2+^-TFSI^−^ complexes become reductively unstable below 0.37 V (vs. Zn/Zn^2+^), which is substantially higher than the reduction potential for the isolated TFSI^−^, corroborating the preferential decomposition of TFSI^−^ over Zn^2+^ reduction.

In support of the above mechanism, X-ray photoelectron spectroscopy (XPS) and Raman analyses were implemented to experimentally probe the existence of the in situ formed interphase. In the F 1s spectra from XPS (Fig. 4b, the C 1s spectrum can be seen in Supplementary Figs. 27a, 28), except for the C–F component arising from the residual Zn(TFSI)_2_ salt, we also observed the presence of ZnF_2_ (684.5 eV) on the cycled Zn^60^, in perfect accordance with the DFT calculation. For the S 2p spectrum shown in Fig. 4c and Supplementary Fig. 27d, a new peak associated with sulfide appears at 161.9 eV^45^, further verifying the decomposition of TFSI^−^. Encouragingly, from the Raman analysis, a strong characteristic peak assigned to ZnF_2_ at 522 cm^−1^ can be clearly detected^61^ (Fig. 4d). On the other hand, the Zn anode cycled in 1 M Zn(TFSI)_2_ displays two obvious Raman peaks at 437 and 563 cm^−1^, related to the formation of ZnO^62^.

FTIR investigations provide additional insights into the chemical features of the Zn-compatible SEI. Compare with pure Zn, the surface of the SEI-coated Zn is enriched with organic functional groups (Supplementary Fig. 30). A blue shift in the C‒F, S=O and C‒N functional groups can be found for the SEI layer (Supplementary Table 5) compared with the ZES electrolyte. Overall, the agreement between spectral characterization and theoretical calculation suggests that the unique anion-containing Zn species in ZES enable TFSI^−^ to reductively decompose and participate in the SEI formation with major components involving ZnF_2_, S/N-rich organic compounds and/or their derivatives. Unlike the passivating layers on the surface of multivalent metals (Zn, Mg, and Ca, etc.)^23,24^, Zn^2+^ is able to penetrate such anion-derived SEI layer, whose ionic conductivity is calculated to be 2.36 × 10^−6^ S cm^−1^ (Supplementary Fig. 31), higher than that of the artificial interphase fabricated on Mg anode (1.19 × 10^−6^ S cm^−1^)^23^. An additional evidence for the effective Zn^2+^ diffusion through SEI can be provided by the low activation energy of 49.7 kJ mol^‒1^ obtained by temperature-dependent impedance measurements (Fig. 4e and Supplementary Fig. 32)^25,63^, which is comparable to that of Li^+^ diffusion across the typical SEI formed in LiPF_6_/EC/EMC (51 kJ mol^‒1^)^25^. Furthermore, several metal fluorides (*M*_*x*_F_*y*_, *M* = Li, Zn, Cu, and Al) have been well acknowledged as main rigid-frame materials for protecting metal anodes, since they can guide the metal nucleation and effectively inhibit the growth of dendrites^64–66^. Meanwhile, the S/N-rich organic compounds could provide sufficient ion channels for Zn^2+^ transport, and their flexibility will accommodate volume changes caused by Zn plating/stripping^67,68^.

In view of the element distribution of the SEI, two additional surface-sensitive techniques, time-of-flight secondary-ion mass spectrometry (TOF-SIMS) and XPS spectra at various sputtering depths were used. As shown in XPS spectra at various sputtering depths (0, 7, and 15 nm) on the SEI accumulated on Zn (Fig. 4f), the signal of metallic Zn at 1044.63 eV can be observed with the depth increased to ca. 15 nm (Fig. 4g and Supplementary Fig. 29), which defines the thickness of SEI layer. Moreover, as the etching depth increased, the intensity of ZnF_2_ increases gradually while those of sulfides and nitrides decrease, indicating that ZnF_2_ mainly exists in the inner SEI region and S/N-rich organic compounds are mainly distributed in the outer SEI layer. Besides, TOF-SIMS results (Fig. 4f and Supplementary Fig. 33) combined with the energy-dispersive spectroscopy (EDS) analyses (Supplementary Fig. 34) further exhibit an even distribution of Zn, F, N, and S elements on the cycled Zn surface, implying the uniformity of the SEI layer.

### The validity of the SEI layer in various electrolyte systems

**Fig. 5:**
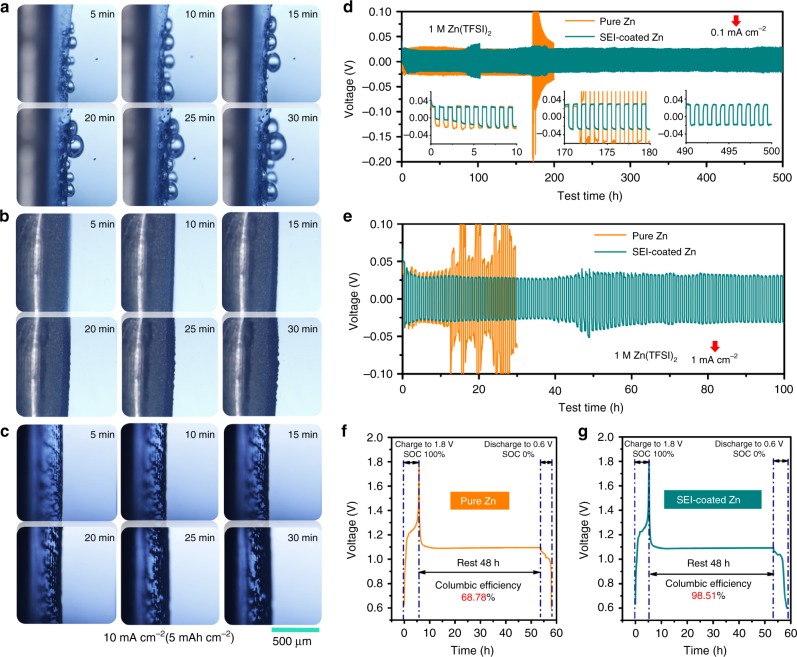
The validity of the SEI layer for Zn electrochemistry. **a**–**c** In situ investigations of the Zn deposition by optical microscopy in Zn/Zn cells. Images of the Zn-electrolyte interface region in **a** 1 M Zn(TFSI)_2_ and **b** ZES. **c** Images of SEI-coated Zn-electrolyte interface region using 1 M Zn(TFSI)_2_. The deposition current density is 10 mA cm^−2^ with the areal capacity of 5 mAh cm^‒2^. Galvanostatic cycling performance of symmetric Zn/Zn cells tests using pure and SEI-coated Zn coupled with 1 M Zn(TFSI)_2_ at **d** low rate of 0.1 mA cm^−2^ (0.05 mAh cm^−2^ for each half cycle) and **e** high rate of 1 mA cm^−2^ (0.5 mAh cm^−2^ for each half cycle). The Zn/V_2_O_5_ cells in 1 M Zn(TFSI)_2_ using **f** pure Zn anode and **g** SEI-coated Zn anode were first fully charged to 1.8 V at 20 mA g^−1^ (based on active materials of cathode), respectively, and then the cells were rested at 100% stage of charge (SOC) for 48 h, followed by full discharging.

More direct evidence for modulated Zn plating/stripping enabled by this SEI was obtained from in situ optical visualization observations of Zn deposition (in a home-made cell, Supplementary Fig. 35a). Not surprisingly, in 1 M Zn(TFSI)_2_, rather uneven Zn electrodeposits and copious air bubbles were observed as early as 5 min after the inception of deposition (Fig. 5a and Supplementary Fig. 35b). For the cell using ZES, uniform and compact Zn deposits can be achieved at capacity of 5 mAh cm^−2^ (10 mA cm^−2^) (Fig. 5b). We further investigated the availability of the SEI layer in the routine aqueous electrolyte. As is shown in Fig. 5c, the gas generation disappears when such SEI-coated Zn anodes obtained in ZES were reassembled in 1 M Zn(TFSI)_2_. Particularly, these surface-modified Zn anode exhibits extended cycling life and lower polarization at both low (0.1 mA cm^−2^) and high (1 mA cm^−2^) rates (Fig. 5d, e). Indeed, Zn surface after deposition remain visibly flat (Supplementary Fig. 35c), which emphasizes a strong correlation between SEI and dendrite-free Zn deposition.

The effect of the SEI on parasitic reactions was evaluated by monitoring the open circuit-voltage decay of fully charged Zn/V_2_O_5_ cells with 1 M Zn(TFSI)_2_ and then discharging after 48 h of storage. 97.8% of the original capacity was retained (Fig. 5f) in cell using SEI-protected Zn anode, exceeding 68.78% using an untreated Zn anode. Obviously, similar to the function of the anode SEI layer obtained in Li-metal anodes, this rigid-flexible coupling SEI formed on Zn surface can eliminate direct contact between the active anode and electrolyte, thereby inhibiting the interface side-reactions (such as H_2_ evolution and passivation) effectively during storage. More importantly, once the SEI forms, the coated Zn surface is functionalized by stable and favorable Zn^2+^ transport with low-diffusion barrier, which can facilitate reversible Zn stripping/plating even applicable for implanted aqueous electrolytes. Note that ZES exhibits lower polarization and better stability for Zn/Zn^2+^ reactions compared with DESs based on other Zn salts (Supplementary Fig. 36), demonstrating the TFSI^−^-induced SEI formation mechanism and the uniqueness of the SEI composition. To the best of our knowledge, the in situ constructed effective SEI has not been reported in ZIBs. This strategy may also be helpful to the development of other MIBs electrolytes that are compatible with their corresponding metal anodes.

### Highly stable ZIBs coupled with cathodes (V_2_O_5_ and Mo_6_S_8_) in the ZES electrolyte

**Fig. 6:**
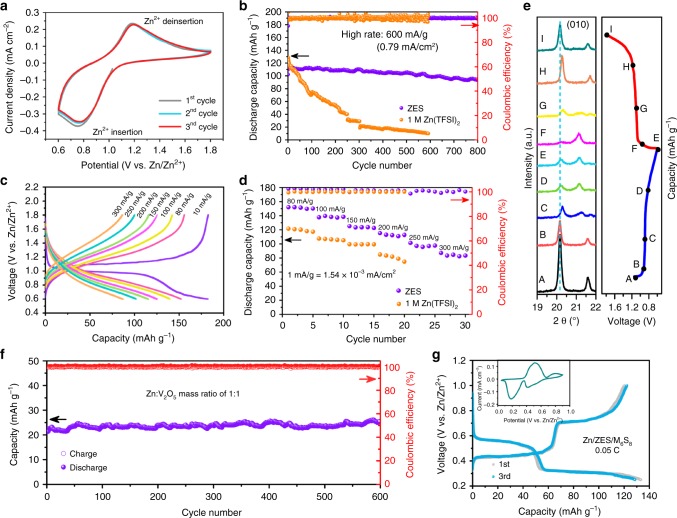
Electrochemical properties of ZIBs. **a** Typical CV curves of the Zn/V_2_O_5_ cell using ZES at a scan rate of 0.5 mV s^−1^. **b** Charge/discharge cycling performance and CE of the Zn/V_2_O_5_ cells with ZES (after activation under 1 A g^−1^) and 1 M Zn(TFSI)_2_ electrolytes at 600 mA g^−1^ (0.79 mA cm^−2^)_._ **c** Charge/discharge curves at various current densities in ZES. **d** Rate performance of ZES and 1 M Zn(TFSI)_2_ electrolytes. **e** XRD patterns of the V_2_O_5_ cathode at different voltage states of the first cycle in ZES (10 mA g^−1^). **f** Long-term cycling performance of the Zn//ZES//V_2_O_5_ cell with the Zn:V_2_O_5_ mass ratio of 1:1 at 8.43 mA cm^−2^ (after activation under same rate; the capacity is calculated based on the total mass of cathode and anode). **g** Typical galvanostatic charge/discharge profiles and CV curves (inset) of the Zn/Mo_6_S_8_ cell with ZES electrolyte. The current densities are calculated on the activated materials of cathode.

Finally, we explored applications of the ZES electrolyte in ZIBs composed of Zn anode and V_2_O_5_ cathode (Fig. 6a). CV profiles of the cell after initial cycle activation are almost overlapped. For comparison, a co-intercalation of Zn^2+^ and hydrated protons (H_3_O^+^) was observed in the cell that contains 1 M Zn(TFSI)_2_ as electrolyte (Supplementary Fig. 37)^69^. From the CV measurement of the Zn/V_2_O_5_ cell at different scan rates, the diffusion-controlled reaction process can be certified in ZES (Supplementary Fig. 38). As expectedly, the cyclic stability of Zn/V_2_O_5_ cells using ZES outperforms their aqueous counterparts at each current density (Fig. 6b and Supplementary Figs. 39, 41). Unlike 1 M Zn(TFSI)_2_, ZES can be cycled without over-charging even at a relatively low rate (10 mA g^−1^) (Supplementary Fig. 40). At a high rate of 200 mA g^−1^ (~34 min rate), the V_2_O_5_ cathode with ZES delivers an excellent stability with a high capacity retention of 91.3% with a high CE ~99.34% for 100 cycles (Supplementary Fig. 41a). Besides, there is only a slight change of the cell overpotential throughout the cycling process (Supplementary Fig. 41b). Considering that the generation of a stabilized SEI extremely relies on the amount of charge passed through the cell^54^, we pre-activated the Zn/V_2_O_5_ cell at 1 A g^−1^ to accelerate the SEI growth on anode, and then tested its long-cycle stability at the rate of 600 mA g^−1^. Such a cell exhibits a highly reversible specific capacity of nearly 110 mAh g^−1^ (based on the mass of V_2_O_5_); 92.8% of initial capacity could be retained over prolonged 800 cycles, along with a high average CE of 99.9% (Supplementary Fig. 42). In sharp contrast, the capacity of the cell with 1 M Zn(TFSI)_2_ rapidly decayed to 61.9 mAh g^−1^ (capacity retention < 50%) after only 150 cycles, which is mainly ascribed to the formation of the insulating passivation layer on Zn anode (Supplementary Fig. 25) that blocks the Zn^2+^ interfacial transport, and the resulting increase in polarization^10,23,26^. The potential of ZES for power-type ZIBs is further evidenced by the attractive rate capability with elevating current density from 80 to 300 mA g^−1^ (Fig. 6c, d). This should be contributed by high active Zn^2+^ diffusion coefficient (1.66 × 10^−6^ cm^2^ s^−1^, Supplementary Fig. 18) and the pseudo-capacitance properties of V_2_O_5_ (Fig. 6c).

Although cycling with a low areal capacity has been demonstrated to assist in maintaining a uniform morphology for metallic anodes^70^, material loadings must be rationally optimized to yield the truly competitive ZIBs for industrial scenarios^16^. Thus, we have attempted to estimate the utility of the ZES electrolyte on a more practical basis by a full cell with a high-mass-loading V_2_O_5_ cathode and a thin Zn foil (20 μm thickness, ~11.7 mAh cm^−2^). When the V_2_O_5_ loading is as high as 14.3 mg cm^−2^, the Zn//ZES//V_2_O_5_ cell can be cycled still shows stable operation over 600 cycles at a high rate of 8.43 mA cm^−2^ with a capacity fading of only 0.0035% cycle^−1^ (the capacity retention of 97.89%) (Fig. 6f). In contrast to most of the previously reported ZIBs, wherein much excessive Zn needs to be used for prolonging the cycle life, the mass ratio between Zn and V_2_O_5_ was set to 1:1 in this cell. Based on the total mass of cathode and anode, the capacity is calculated to be 25.5 mAh g^−1^, corresponding to an energy density of 25.8 Wh kg^−1^. In addition, further reducing the Zn:V_2_O_5_ mass ratio to 0.5:1 can provide an improved energy density of 40.9 Wh kg^−1^ (Supplementary Fig. 43). In the case of development of the Zn^2+^-storage cathodes taking into account stability, capacity and operation voltage simultaneously, there is still vast scope for improvements in energy density of ZES-based ZIBs^71^. The ex situ XRD test (Fig. 6e and Supplementary Fig. 44) shows that a highly stable and completely reversible structure evolution occurred on the V_2_O_5_ cathode during charge/discharge processes in ZES. A Zn/Mo_6_S_8_ cell was assembled to demonstrate the versatility of ZES for ZIB applications (Fig. 6g). Such a cell delivers a high discharge capacity of 128.6 mAh g^−1^ (based on the mass of Mo_6_S_8_) after three cycles, which is close to the theoretical value of Mo_6_S_8_ (129 mAh g^−1^)^72^. Two pairs of typical redox peaks correspond well to the two-step Zn^2+^ (de-)intercalation processes, analogous to cases of previously reported^72^.

## Discussion

In summary, we have demonstrated that the established in situ SEI protection is a feasible strategy toward rechargeable Zn-metal anodes. Due to the direct coordination between cations and anions in a form of large-size cationic complexes endowed by the ZES, the reductive decomposition of TFSI^−^ is induced before the Zn deposition during the initial cycling process, allowing a well-defined Zn-compatible SEI layer with a rich content of mechanically rigid ZnF_2_ and Zn^2+^-permeable organic components. With this interface modulation, dendrite-free and intrinsically stable Zn plating/stripping can be realized at the areal capacity of >2.5 mAh cm^−2^ or even under a common dilute aqueous electrolyte system. Zn//ZES//V_2_O_5_ cells present remarkable electrochemical reversibility (an average CE of ~99.9%, superior to most aqueous ZIBs^9,73,74^) and laudable capacity retention even under rigorous but practically desirable cathode-anode loading conditions. Given the extendibility of this strategy, we envision that this study will provide an unprecedented avenue for tackling the dilemmas raised by the intrinsic properties of multivalent metal anodes, which may lead to the potential fabrication of energy-storage devices.

## Methods

### Preparation of electrolytes and cathodes

The ZES samples were formed by readily mixing the two components (Zn(TFSI)_2_ and Ace) with the required molar ratios (the Zn(TFSI)_2_/Ace molar ratio between 1:4 and 1:9) at room temperature (Supplementary Fig. 1). Homogenous and transparent liquids can be obtained directly after heating the mixtures at 80 °C with gentle stirring 2 h. Subsequently, the electrolytes were stored in a dry atmosphere for further use. The micro-sized V_2_O_5_ material was purchased from Aldrich. The V_2_O_5_ electrodes used here comprise 70 wt% V_2_O_5_, 20 wt% Super P carbon, and 10 wt% polyvinylidene fluoride (PVDF; Sigma). Mo_6_S_8_ (Chevrel phase) was synthesized according to the previously reported method^72^. The Mo_6_S_8_ electrodes were prepared by the same procedure, but Mo_6_S_8_ (80 wt%), Super P carbon (10 wt%), and PVDF (10 wt%), which were mixed and dispersed in N-methyl-2-pyrrolidone and cast on to the Ti current collector (10 μm in thickness). V_2_O_5_ and Mo_6_S_8_ cathodes were punched in the diameter of 1.2 cm (1.1304 cm^2^) for the full cell tests. The active mass loading for the V_2_O_5_ cathode materials is 1.6 ± 1 mg cm^−2^ for normal tests, while that for the Mo_6_S_8_ cathode is ∼1.5 mg cm^−2^. The high active mass loading for the V_2_O_5_ cathode materials is 14.3 mg cm^−2^ (4.1 mAh cm^−2^, 290 mAh g^−1^ for the V_2_O_5_) and the thicknesses of Zn foils are 20 and 10 μm (14.28 and 7.14 mg cm^−2^, respectively) for the practical utility evaluation test.

### Electrochemical measurements

EIS was performed by an electrochemical working station (VMP-300) over the frequency range 0.1–7 × 10^6^ Hz with a perturbation amplitude of 5 mV to better investigate the interfacial stability between Zn metal and different electrolytes. Electrochemical cycling tests in Zn/Zn symmetric cells, Ti/Zn cells, Zn/V_2_O_5_ and Zn/Mo_6_S_8_ cells were conducted in CR2032-type coin cells with LAND testing systems. All cells were assembled in an open environment and a glass fiber with a diameter of 16.5 mm was used as the separator.

### Characterization

SEM (Hitachi S-4800) was employed to detect the morphologies of Zn deposits on the Zn-metal anodes or the Ti foils. FTIR measurements were carried out on a Perkin-Elmer spectrometer in the transmittance mode. XRD patterns were recorded in a Bruker-AXS Micro-diffractometer (D8 ADVANCE) with Cu-K_α1_ radiation (*λ* = 1.5405 Å). Raman spectra were recorded at room temperature using a Thermo Scientific DXRXI system with excitation from an Ar laser at 532 nm. A differential scanning calorimeter (TA, dsc250) was used to evaluate the thermal properties of the electrolytes. Samples are scanned from −80–100 °C at a rate of 5 °C–min^−1^ under a nitrogen atmosphere. An in situ optical microscope from the Olympus Corporation was used to observe the depositional morphology of Zn with different electrolytes in real time in order to study the interfacial stability. XPS was performed on a Thermo Scientific ESCA Lab 250Xi to characterize the surface components. TOF-SIMS (Germany, TOF-SIMS5) was employed to measure the components as a function of depth.

### Calculation methods

All quantum chemical calculations were performed by applying the DFT method with the B3LYP level and 6–31+G (d, p) basis set using Gaussian 09 program package. The structural optimization was determined by minimizing the energy without imposing molecular symmetry constraints. The binding energy of the anion-containing Zn species were defined as the interaction between different molecule fragments, composed of the interaction between Zn^2+^, TFSI^−^ and Ace. The binding energy *E* was calculated according to Eq. (1), the expression as follows:

1 $$E = E_{{\mathrm{total}}} - nE(X)$$

where *E*_total_ is the structure total energy, *E*(*X*) is the energy of different molecule fragments (*X* = Zn^2+^, TFSI^−^, Ace), and *n* is the number of corresponding molecule fragments according to the different structure configurations

The reduction potentials for the TFSI^−^ anion with different paths in the solution containing Zn^2+^ were calculated according to Eq. (2), in which the values of the reduction potentials were converted to the Zn/Zn^2+^ scale by subtraction of 3.66 V as discussed extensively elsewhere^42,75^

2 $$E^0 = - \frac{{{\mathrm{\Delta }}G_{298{\mathrm{K}}}^0}}{{nF}} - 3.66\;{\mathrm{V}}$$

where ${\mathrm{\Delta }}G_{298{\mathrm{K}}}^0$ is the Gibbs energy of the reduction reaction of different paths, *n* is the transferred electron number, *F* is Faraday constant.

## Supplementary information

Supplementary Information

Peer Review File

## Data Availability

The datasets generated during the current study are included in this published article (and its supplementary information files) are available from the corresponding author on reasonable request.

## References

[1] Zhang N (2017). Rechargeable aqueous zinc-manganese dioxide batteries with high energy and power densities. Nat. Commun..

[2] Canepa P (2017). Odyssey of multivalent cathode materials: open questions and future challenges. Chem. Rev..

[3] Ponrouch A, Frontera C, Barde F, Palacin MR (2016). Towards a calcium-based rechargeable battery. Nat. Mater..

[4] Muldoon J, Bucur CB, Gregory T (2014). Quest for nonaqueous multivalent secondary batteries: magnesium and beyond. Chem. Rev..

[5] Aurbach D (2000). Prototype systems for rechargeable magnesium batteries. Nature.

[6] Caramia V, Bozzini B (2014). Material science aspects of zinc-air batteries: a review. Mater. Renew. Energy.

[7] Wang F (2018). How water accelerates bivalent ion diffusion at the electrolyte/electrode interface. Angew. Chem. Int. Ed..

[8] Song M, Tan H, Chao D, Fan HJ (2018). Recent advances in Zn-ion batteries. Adv. Funct. Mater..

[9] Fang G, Zhou J, Pan A, Liang S (2018). Recent advances in aqueous zinc-ion batteries. Acs Energy Lett..

[10] Lu W, Xie C, Zhang H, Li X (2018). Inhibition of zinc dendrite growth in zinc-based batteries. Chemsuschem.

[11] Naveed A, Yang H, Yang J, Nuli Y, Wang J (2019). Highly reversible and rechargeable safe Zn batteries based on a triethyl phosphate electrolyte. Angew. Chem. Int. Ed..

[12] Zhao J (2019). “Water-in-deep eutectic solvent” electrolytes enable zinc metal anodes for rechargeable aqueous batteries. Nano Energy.

[13] Kang L (2018). Nanoporous CaCO_3_ coatings enabled uniform Zn stripping/plating for long-life zinc rechargeable aqueous batteries. Adv. Energy Mater..

[14] Parker JF (2017). Rechargeable nickel-3D zinc batteries: an energy-dense, safer alternative to lithium-ion. Science.

[15] Gupta T (2016). Improving the cycle life of a high-rate, high-potential aqueous dual ion battery using hyper-dendritic zinc and copper hexacyanoferrate. J. Power Sources.

[16] Wang F (2018). Highly reversible zinc metal anode for aqueous batteries. Nat. Mater..

[17] Chen CY, Matsumoto K, Kubota K, Hagiwara R, Xu Q (2019). A room-temperature molten hydrate electrolyte for rechargeable zinc-air batteries. Adv. Energy Mater..

[18] Wu X (2019). Reverse dual-ion battery via a ZnCl_2_ water-in-salt electrolyte. J. Am. Chem. Soc..

[19] Xu W (2014). Lithium metal anodes for rechargeable batteries. Energy Environ. Sci..

[20] Zhang XQ (2019). Regulating anions in the solvation sheath of lithium ions for stable lithium metal batteries. Acs Energy Lett..

[21] Ren XD (2018). Guided lithium metal deposition and improved lithium coulombic efficiency through synergistic effects of LiAsF_6_ and cyclic carbonate additives. Acs Energy Lett..

[22] Suo L (2017). How solid-electrolyte interphase forms in aqueous electrolytes. J. Am. Chem. Soc..

[23] Son SB (2018). An artificial interphase enables reversible magnesium chemistry in carbonate electrolytes. Nat. Chem..

[24] Wang D (2017). Plating and stripping calcium in an organic electrolyte. Nat. Mater..

[25] Zhang XQ (2018). Highly stable lithium metal batteries enabled by regulating the solvation of lithium ions in nonaqueous electrolytes. Angew. Chem. Int. Ed..

[26] Mainar AR (2018). An overview of progress in electrolytes for secondary zinc-air batteries and other storage systems based on zinc. J. Energy Storage.

[27] Smith EL, Abbott AP, Ryder KS (2014). Deep eutectic solvents (DESs) and their applications. Chem. Rev..

[28] Zhang QH, Vigier KD, Royer S, Jerome F (2012). Deep eutectic solvents: syntheses, properties and applications. Chem. Soc. Rev..

[29] Yamada Y, Wang J, Ko S, Watanabe E, Yamada A (2019). Advances and issues in developing salt-concentrated battery electrolytes. Nat. Energy.

[30] Abbott AP, Barron JC, Ryder KS, Wilson D (2007). Eutectic-based ionic liquids with metal-containing anions and cations. Chem. Eur. J..

[31] Abbott AP, Capper G, Davies DL, Rasheed R (2004). Ionic liquids based upon metal halide/substituted quaternary ammonium salt mixtures. Inorg. Chem..

[32] Zheng B (2018). Stabilizing Li_10_SnP_2_S_12_/Li interface via an in situ formed solid electrolyte interphase layer. ACS Appl. Mater. Interfaces.

[33] Borodin O, Smith GD (2006). LiTFSI structure and transport in ethylene carbonate from molecular dynamics simulations. J. Phys. Chem. B.

[34] Tsuzuki S, Tokuda H, Hayamizu K, Watanabe M (2005). Magnitude and directionality of interaction in ion pairs of ionic liquids: Relationship with ionic conductivity. J. Phys. Chem. B.

[35] He M, Fic K, Frąckowiak E, Novák P, Berg EJ (2016). Influence of aqueous electrolyte concentration on parasitic reactions in high-voltage electrochemical capacitors. Energy Storage Mater..

[36] Henderson WA (2006). Glyme-lithium salt phase behavior. J. Phys. Chem. B.

[37] Seo DM, Borodin O, Han S-D, Boyle PD, Henderson WA (2012). Electrolyte solvation and ionic association II. Acetonitrile-lithium salt mixtures: highly dissociated salts. J. Electrochem. Soc..

[38] Liang HY (2001). New binary room-temperature molten salt electrolyte based on urea and LiTFSI. J. Phys. Chem. B.

[39] Hu YS, Wang ZX, Li H, Huang XJ, Chen LQ (2005). Spectroscopic studies on the cation-anion, cation-solvent and anion-solvent interactions in the LiCF_3_SO_3_/acetamide complex system. Spectrochim. Acta A.

[40] Yu ZX (2010). A series of LiI/acetamide phase transition electrolytes and their applications in dye-sensitized solar cells. Electrochim. Acta.

[41] Narayanan NSV, Ashokraj BV, Sampath S (2009). Physicochemical, electrochemical, and spectroscopic characterization of zinc-based room-temperature molten electrolytes and their application in rechargeable batteries. J. Electrochem. Soc..

[42] Suo L (2015). “Water-in-salt” electrolyte enables high-voltage aqueous lithium-ion chemistries. Science.

[43] Bakker A, Gejji S, Lindgren J, Hermansson K, Probst MM (1995). Contact ion pair formation and ether oxygen coordination in the polymer electrolytes M[N(CF_3_SO_2_)_2_]_2_PEO*n* for M = Mg, Ca, Sr and Ba. Polymer.

[44] Bakker A, Lindgren J, Hermansson K (1996). Polymer electrolytes based on triblock-copoly(oxyethylene/oxypropylene/oxyethylene) systems. Polymer.

[45] Yamada Y (2014). Unusual stability of acetonitrile-based superconcentrated electrolytes for fast-charging lithium-ion batteries. J. Am. Chem. Soc..

[46] Zhou Q (2011). Phase behavior of ionic liquid-LiX mixtures: pyrrolidinium cations and TFSI^−^ anions-linking structure to transport properties. Chem. Mater..

[47] Dye JL, Faber MP, Karl DJ (1960). Transference numbers and conductances of multivalent salts in aqueous solution: zinc sulfate and zinc perchlorate. J. Am. Chem. Soc..

[48] Plancha, M. J. C. Characterisation and modelling of multivalent polymer electrolytes. *Polym. Electrolytes* 340–377 (2010).

[49] Ueno K (2015). Li^+^ solvation and ionic transport in lithium solvate ionic liquids diluted by molecular solvents. J. Phys. Chem. C..

[50] Simons TJ, MacFarlane DR, Forsyth M, Howlett PC (2014). Zn electrochemistry in 1-ethyl-3-methylimidazolium and N-butyl-N-methylpyrrolidinium dicyanamides: promising new rechargeable Zn battery electrolytes. Chemelectrochem.

[51] Xu M, Ivey DG, Qu W, Xie Z (2014). Improved Zn/Zn(II) redox kinetics, reversibility and cyclability in 1-ethyl-3-methylimmidazolium dicyanamide with water and dimethyl sulfoxide added. J. Power Sources.

[52] Tarascon JM, Armand M (2001). Issues and challenges facing rechargeable lithium batteries. Nature.

[53] Suo L, Hu YS, Li H, Armand M, Chen L (2013). A new class of solvent-in-salt electrolyte for high-energy rechargeable metallic lithium batteries. Nat. Commun..

[54] Adams BD, Zheng J, Ren X, Xu W, Zhang JG (2018). Accurate determination of coulombic efficiency for lithium metal anodes and lithium metal batteries. Adv. Energy Mater..

[55] Liu Z (2016). Dendrite-free nanocrystalline zinc electrodeposition from an ionic liquid containing nickel triflate for rechargeable Zn-based batteries. Angew. Chem. Int. Ed..

[56] Chen S (2018). High-voltage lithium-metal batteries enabled by localized high-concentration electrolytes. Adv. Mater..

[57] Zhang N (2016). Cation-deficient spinel ZnMn_2_O_4_ cathode in Zn(CF_3_SO_3_)_2_ electrolyte for rechargeable aqueous Zn-ion battery. J. Am. Chem. Soc..

[58] Zhang C (2018). A ZnCl_2_ water-in-salt electrolyte for a reversible Zn metal anode. Chem. Commun..

[59] Suo L (2018). Fluorine-donating electrolytes enable highly reversible 5-V-class Li metal batteries. Proc. Natl Acad. Sci. USA.

[60] Gaarenstroom SW, Winograd N (1977). Initial and final-state effects in esca spectra of cadmium and silver-oxides. J. Chem. Phys..

[61] Porto SPS, Fleury PA, Damen TC (1967). Raman spectra of TiO_2_, MgF_2_, ZnF_2_, FeF_2_, and MnF_2_. Phys. Rev..

[62] Damen TC, Porto SPS, Tell B (1966). Raman effect in zinc oxide. Phys. Rev..

[63] Xu K, Lam Y, Zhang SS, Jow TR, Curtis TB (2007). Solvation sheath of Li^+^ in nonaqueous electrolytes and its implication of graphite/electrolyte interface chemistry. J. Phys. Chem. C..

[64] Jia W (2019). ZnF_2_ coated three dimensional Li-Ni composite anode for improved performance. J. Materiomics.

[65] Zhao Q, Liu X, Stalin S, Khan K, Archer LA (2019). Solid-state polymer electrolytes with in-built fast interfacial transport for secondary lithium batteries. Nat. Energy.

[66] Yan C (2018). An armored mixed conductor interphase on a dendrite-free lithium-metal anode. Adv. Mater..

[67] Yu H (2017). Dendrite-free lithium deposition with self-aligned columnar structure in a carbonate–ether mixed electrolyte. Acs Energy Lett..

[68] Yan C (2018). Dual-layered film protected lithium metal anode to enable dendrite-free lithium deposition. Adv. Mater..

[69] Sun W (2017). Zn/MnO_2_ battery chemistry with H^+^ and Zn^2+^ coinsertion. J. Am. Chem. Soc..

[70] Louli AJ (2019). Exploring the impact of mechanical pressure on the performance of anode-free lithium metal cells. J. Electrochem. Soc..

[71] Chen L (2019). Achieving high energy density through increasing the output voltage: a highly reversible 5.3 V battery. Chem.

[72] Chae MS, Heo JW, Lim S-C, Hong S-T (2016). Electrochemical zinc-ion intercalation properties and crystal structures of ZnMo_6_S_8_ and Zn_2_Mo_6_S_8_ chevrel phases in aqueous electrolytes. Inorg. Chem..

[73] Li W, Wang K, Cheng S, Jiang K (2018). A long-life aqueous Zn-ion battery based on Na_3_V_2_(PO_4_)_2_F_3_ cathode. Energy Storage Mater..

[74] Zhang L, Chen L, Zhou X, Liu Z (2015). Towards high-voltage aqueous metal-ion batteries beyond 1.5 V: the zinc/zinc hexacyanoferrate system. Adv. Energy Mater..

[75] Borodin O, Behl W, Jow TR (2013). Oxidative stability and initial decomposition reactions of carbonate, sulfone, and alkyl phosphate-based electrolytes. J. Phys. Chem. C..

