# Supplementary material for: Zinc anode-compatible in-situ solid electrolyte interphase via cation solvation modulation
Source: Nat Commun. 2019 Nov 26;10:5374. doi: 10.1038/s41467-019-13436-3 (PMC6879498; doi:10.1038/s41467-019-13436-3)
Supplement: Supplementary file 1 — Supplementary Information [file 41467_2019_13436_MOESM1_ESM.pdf]

Supplementary Information

Zinc Anode-Compatible In-Situ Solid Electrolyte Interphase via Cation Solvation Modulation

Huayu Qiu et al.

Supplementary Figures

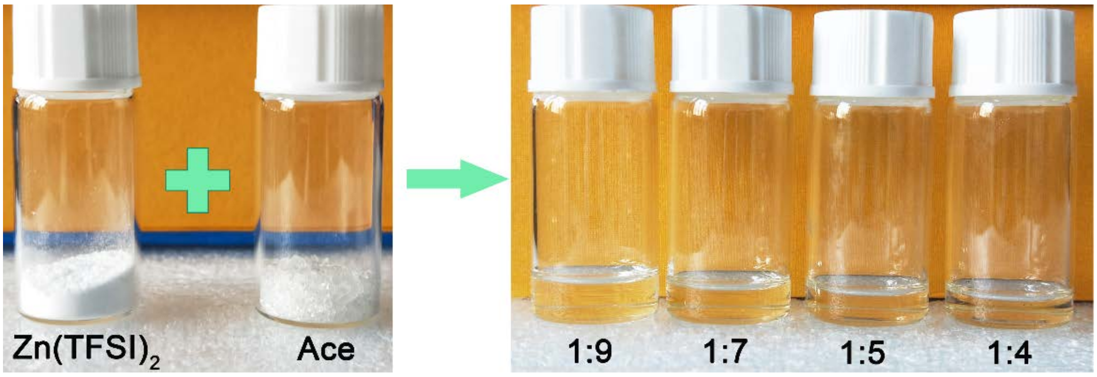

Supplementary Fig. 1 Scheme for preparing the ZESs. Preparation the ZESs in a predetermined $Zn(TFSI)_2$/Ace molar ratio range (1:9−1:4).

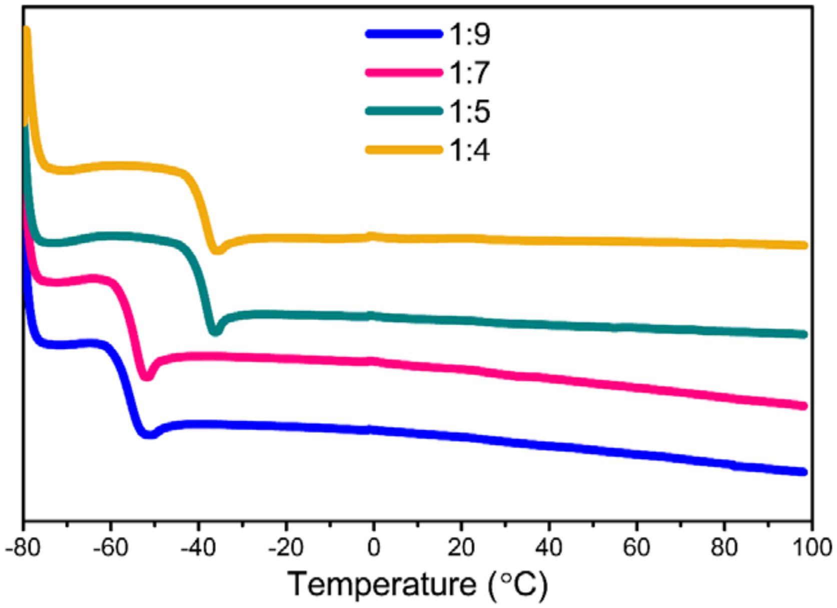

Supplementary Fig. 2 DSC results for ZESs with different Zn(TFSI)$_2$/Ace molar ratios (1:9–1:4).

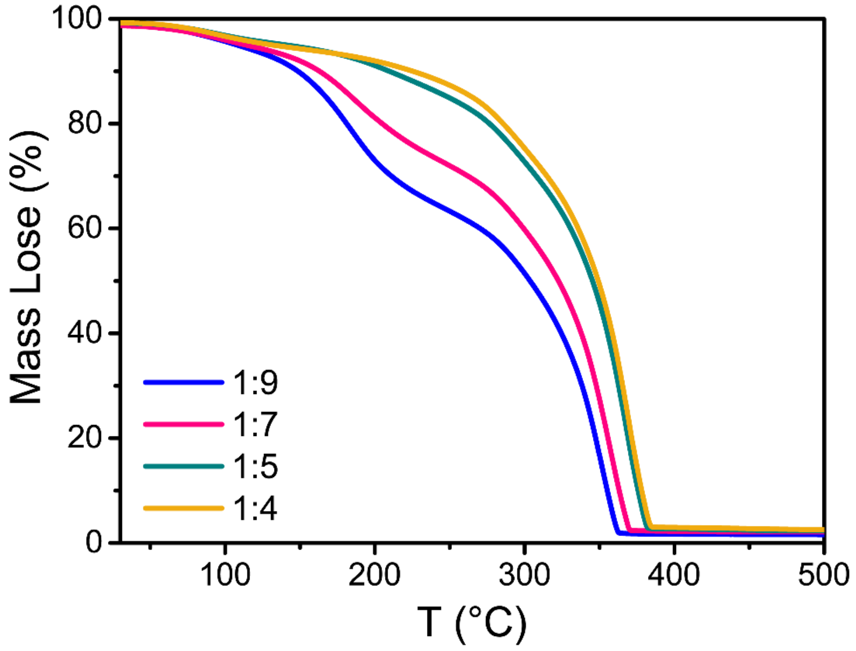

Supplementary Fig. 3 Thermo gravimetric analysis (TGA) of ZESs with different Zn(TFSI)$_2$/Ace molar ratios (1:9−1:4).

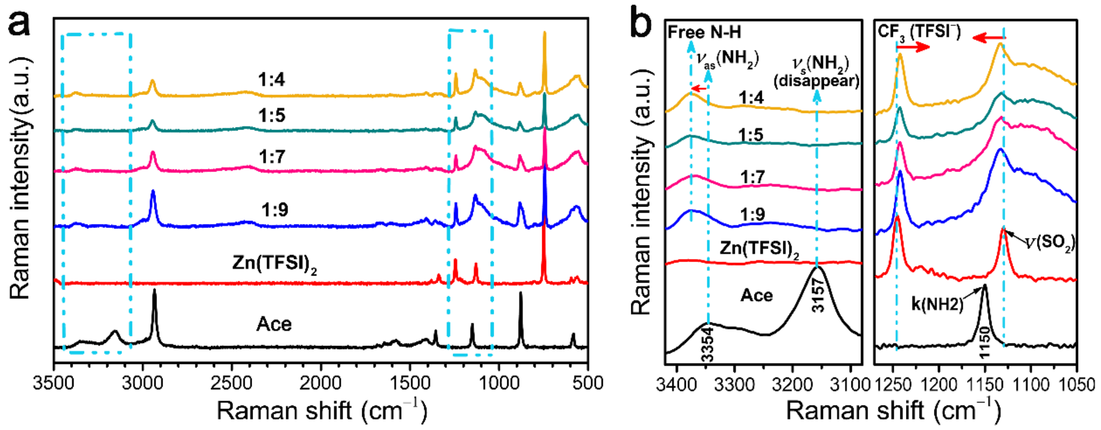

Supplementary Fig. 4 Raman spectra of ZESs. (a) Raman spectra of ZESs with different $Zn(TFSI)_2$/Ace molar ratios (1:9−1:4). **(b)** Partial enlargement view of **(a)**.

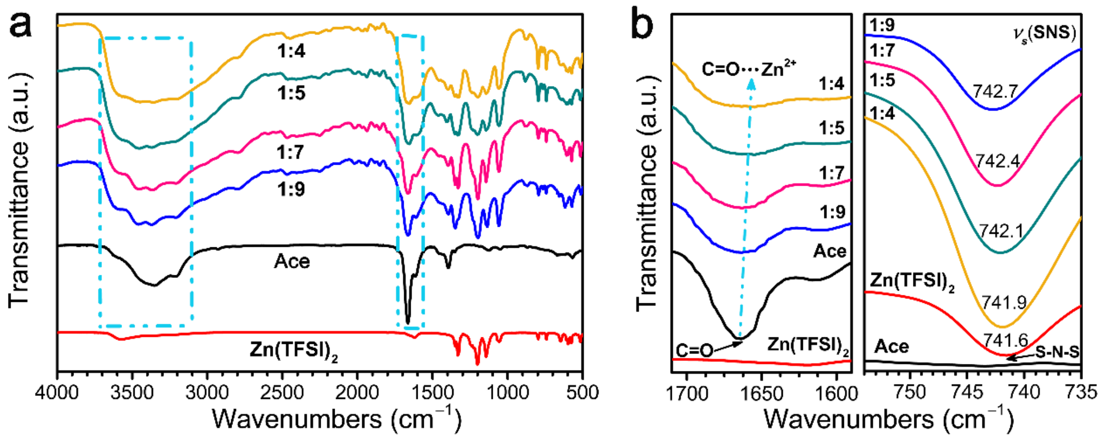

Supplementary Fig. 5 FTIR spectra of ZESs. (a) FTIR spectra of ZESs with different $Zn(TFSI)_2$/Ace molar ratios (1:9−1:4). **(b)** Partial enlargement view of **(a)**.

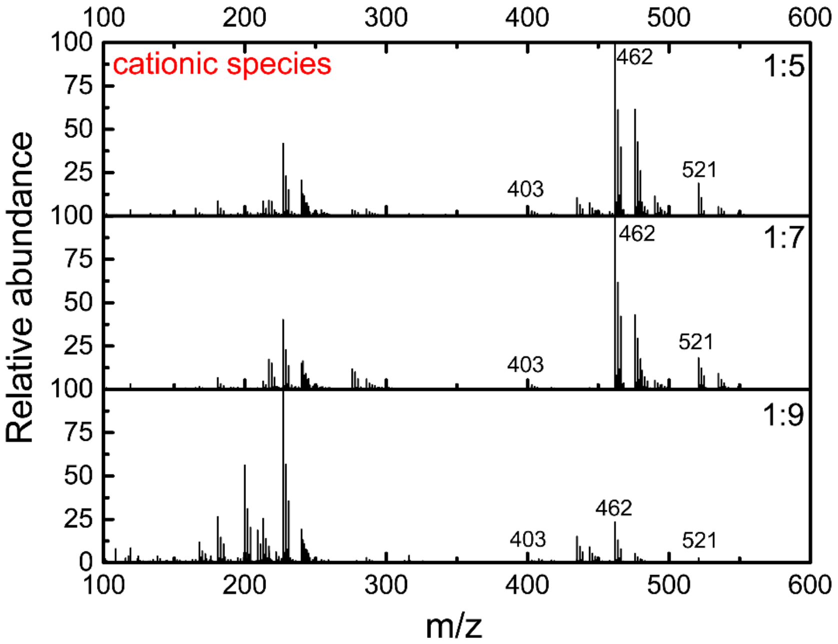

Supplementary Fig. 6 Cationic Zn complexes in ZESs. Cationic high resolution mass spectra (HRMS) of ZESs with different $Zn(TFSI)_2$/Ace molar ratios (1:9, 1:7 and 1:5), showing the presence of a number of cationic Zn complexes, including $[ZnTFSI(Ace)]^+$ (m/z 403), $[ZnTFSI(Ace)_2]^+$ (m/z 462) and $[ZnTFSI(Ace)_3]^+$ (m/z 521).

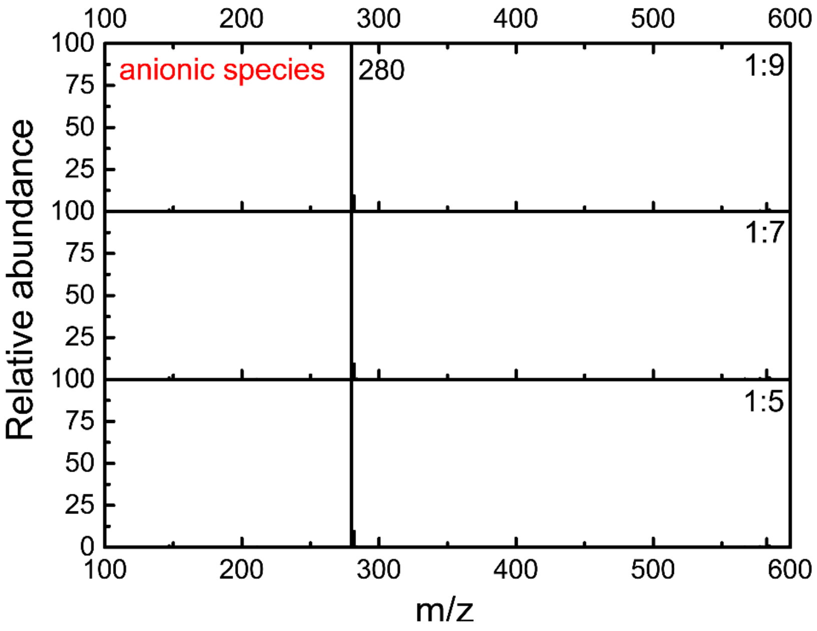

Supplementary Fig. 7 Anionic species in ZESs. Anionic HRMS of ZESs with different $Zn(TFSI)_2$/Ace molar ratios (1:9, 1:7 and 1:5). The only anionic species was found to be $TFSI^-$.

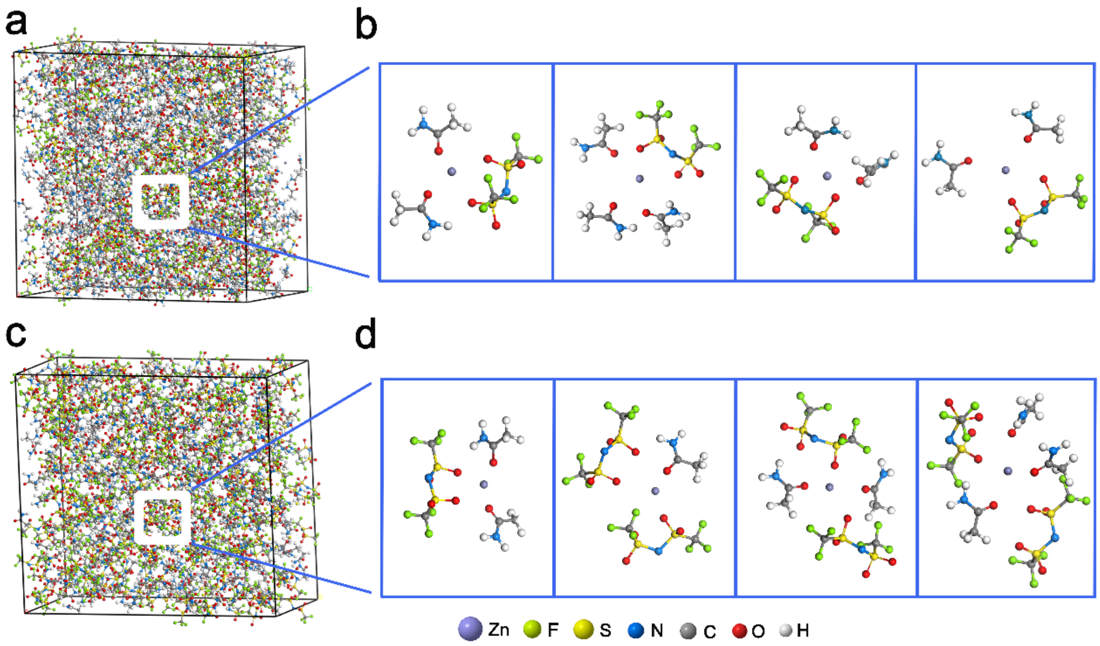

Supplementary Fig. 8 MD simulations of the Zn^{2+}-solvation structure. Snapshots of the MD simulation boxes for ZESs with $Zn(TFSI)_2$/Ace molar ratios of **(a)** 1:7 and **(c)** 1:4 at 353 K. Representative Zn^{2+}-solvation structures for **(b)** 1:7 and **(d)** 1:4.

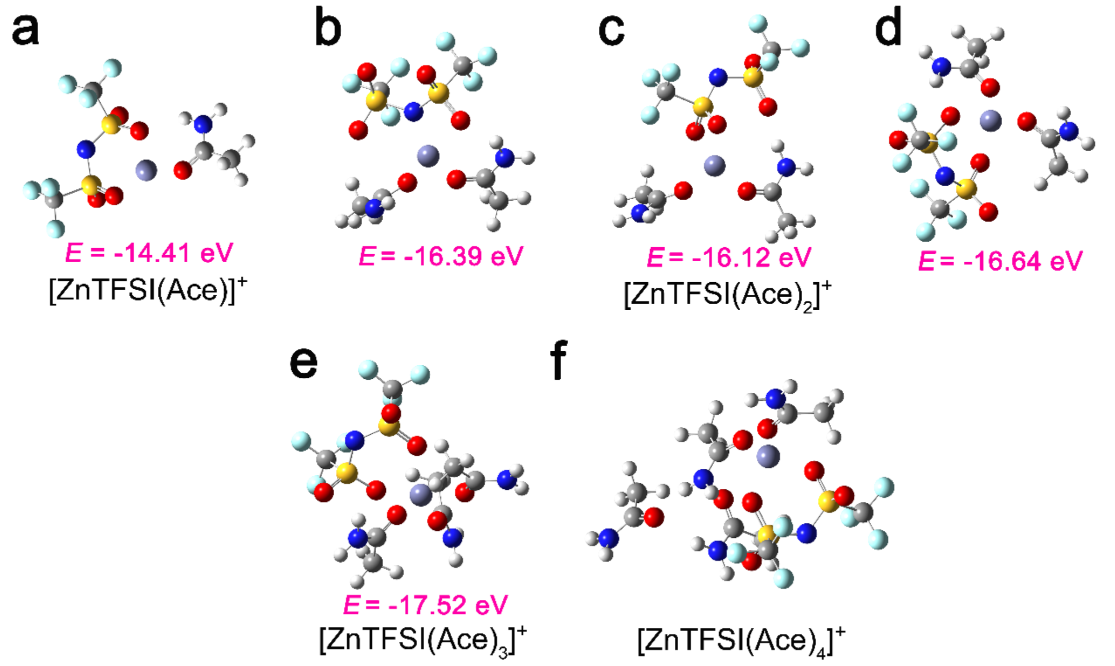

Supplementary Fig. 9 The DFT geometry optimization of the $[ZnTFSI(Ace)_n]^+$ complexes. Optimized geometries of **(a)** $[ZnTFSI(Ace)]^+$ (C_1-O-Π), **(b)** $[ZnTFSI(Ace)_2]^+$ (C_1-O-N-I), (c) $[ZnTFSI(Ace)_2]^+$ (C_1-O-Π), **(d)** $[ZnTFSI(Ace)_2]^+$ (C_2-O-Π), **(e)** $[ZnTFSI(Ace)_3]^+$ (C_1-O-Π), **(f)** $[ZnTFSI(Ace)_4]^+$ (C_1-O-I) based on DFT calculations (Zn-purple, N-blue, O-red, S-yellow, F-light blue, C-light gray, H-white). E: the total binding energy between Zn^{2+}, $TFSI^-$ and Ace. The $TFSI^-$ anion is known to have two different low-energy conformational states: a *cisoid* form (C_1) with the CF_3 groups on the same side of the S–N–S plane and a *transoid* form (C_2) with the CF_3 groups on opposite sides of the plane[1]. I, monodentate coordinated $TFSI^-$ anions; Π, bidentate coordinated $TFSI^-$ anions.

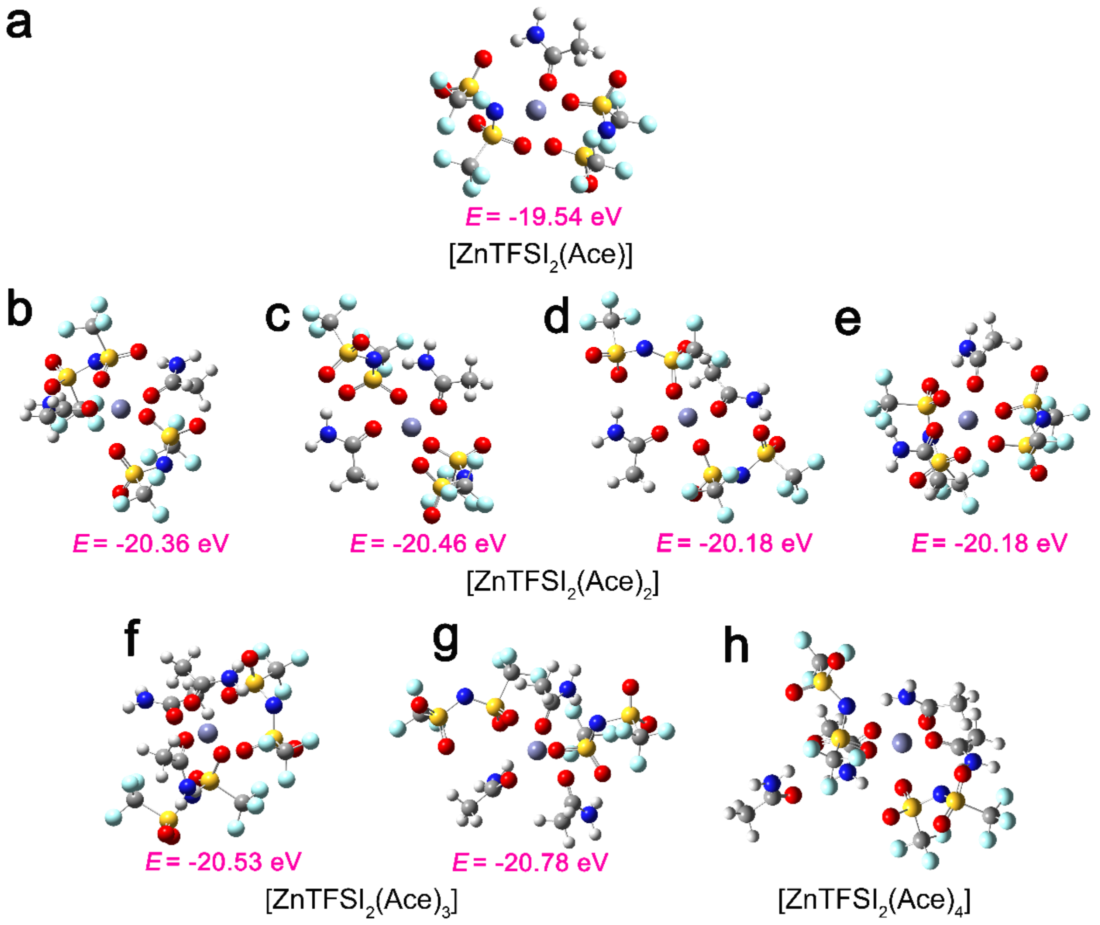

Supplementary Fig. 10 The DFT geometry optimization of the [ZnTFSI$_2$(Ace)$_n$]$^+$ complexes. Optimized geometries of the **(a)** [ZnTFSI$_2$(Ace)$_2$] (C_1-O-I, C_1-O-Π), **(b)** [ZnTFSI$_2$(Ace)$_2$] (C_1-O-Π, C_2-O-I), **(c)** [ZnTFSI$_2$(Ace)$_2$] (C_1-O-I, C_2-O-Π), **(d)** [ZnTFSI$_2$(Ace)$_2$] (C_1-O-I, C_1-O-I), **(e)** [ZnTFSI$_2$(Ace)$_2$] (C_1-O-Π, C_2-O-Π), **(f)** [ZnTFSI$_2$(Ace)$_3$] (C_1-O-Π, C_2-O-I), **(g)** [ZnTFSI$_2$(Ace)$_3$] (C_2-O-I, C_2-O-I), **(h)** [ZnTFSI$_2$(Ace)$_4$] (C_1-O-I, C_2-O-I) based on DFT calculations (Zn-purple, N-blue, O-red, S-yellow, F-light blue, C-light gray, H-white).

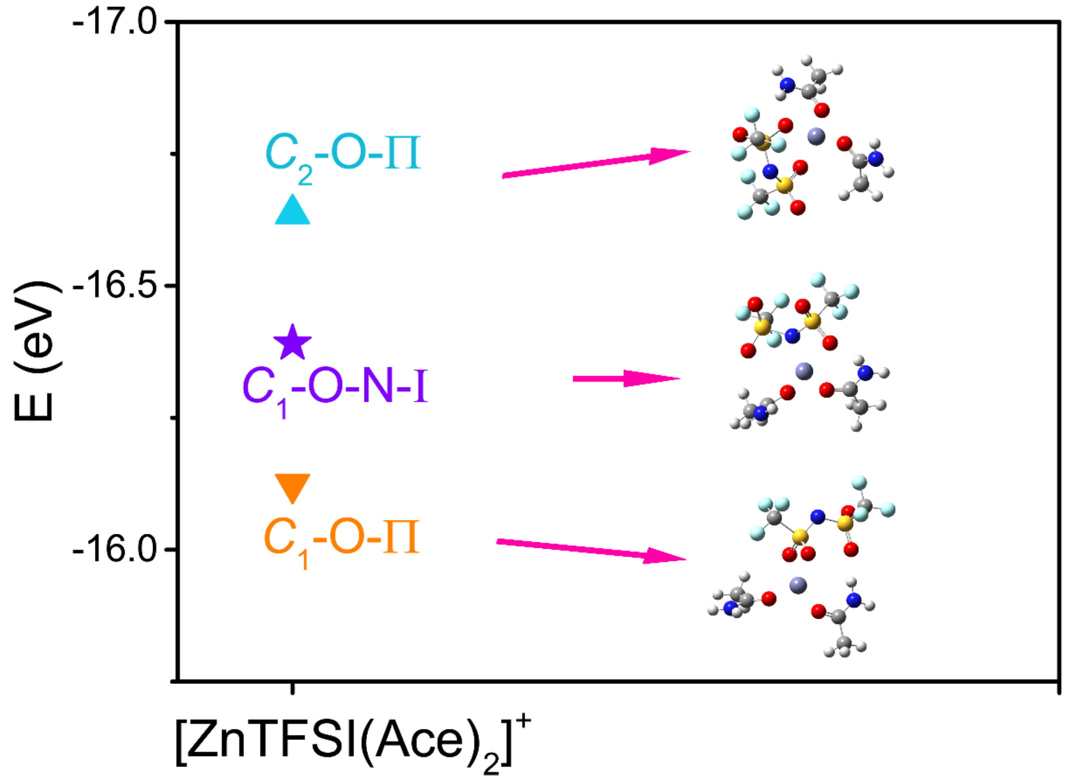

Supplementary Fig. 11 The total interaction energy (*E*) of $[ZnTFSI(Ace)_2]^+$ with three different geometries (Zn-purple, N-blue, O-red, S-yellow, F-light blue, C-light gray, H-white).

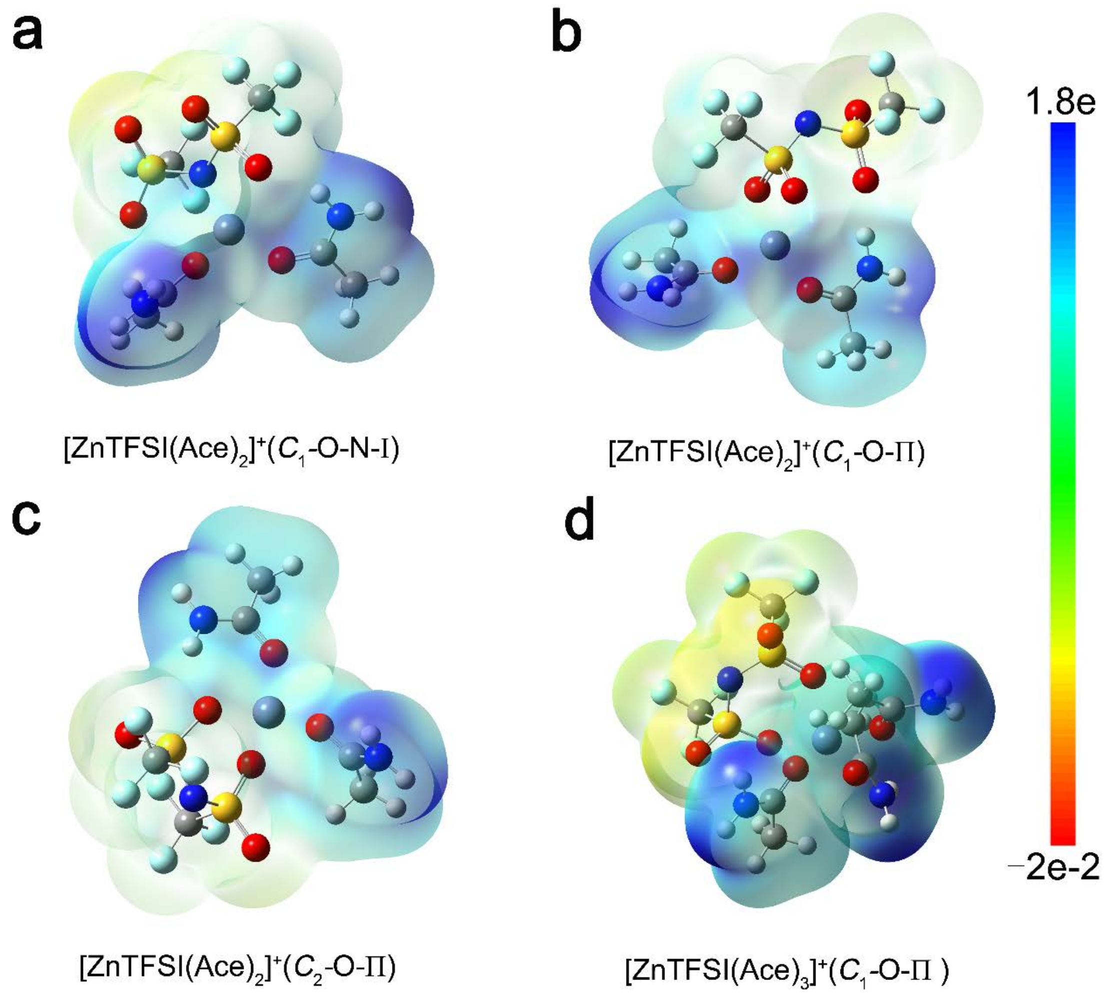

Supplementary Fig. 12 The molecular electrostatic potential energy surface of the $[ZnTFSI(Ace)_n]^+$ (n = 2 and 3) species. Electron density from total SCF density (isoval = 0.001).

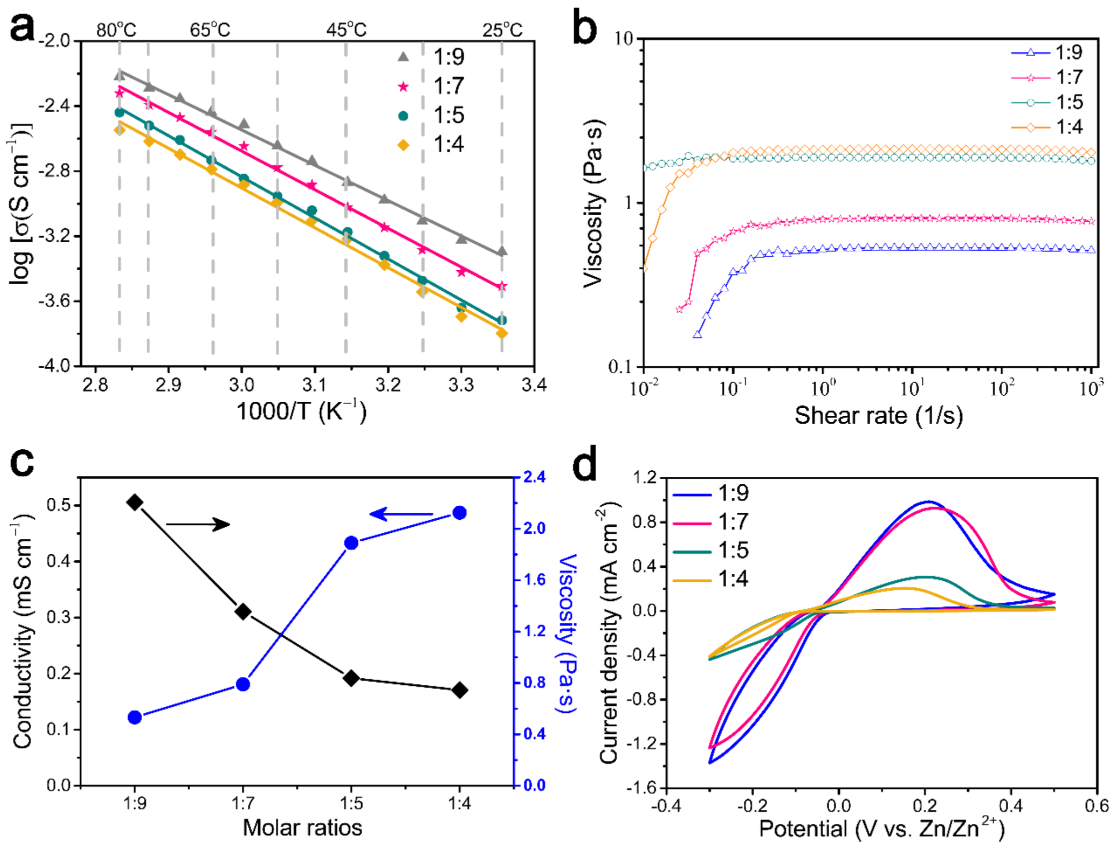

Supplementary Fig. 13 Characterization of ZESs with different Zn(TFSI)$_2$/Ace molar ratios (1:9−1:4). (a) The Arrhenius plot of the ion conductivity. **(b)** The viscosity changing with time measured by rheometer. **(c)** Variation of viscosity and conductivity (25 °C). **(d)** CV curves of Zn/SS cells at a scanning rate of 1 mV s^{-1}.

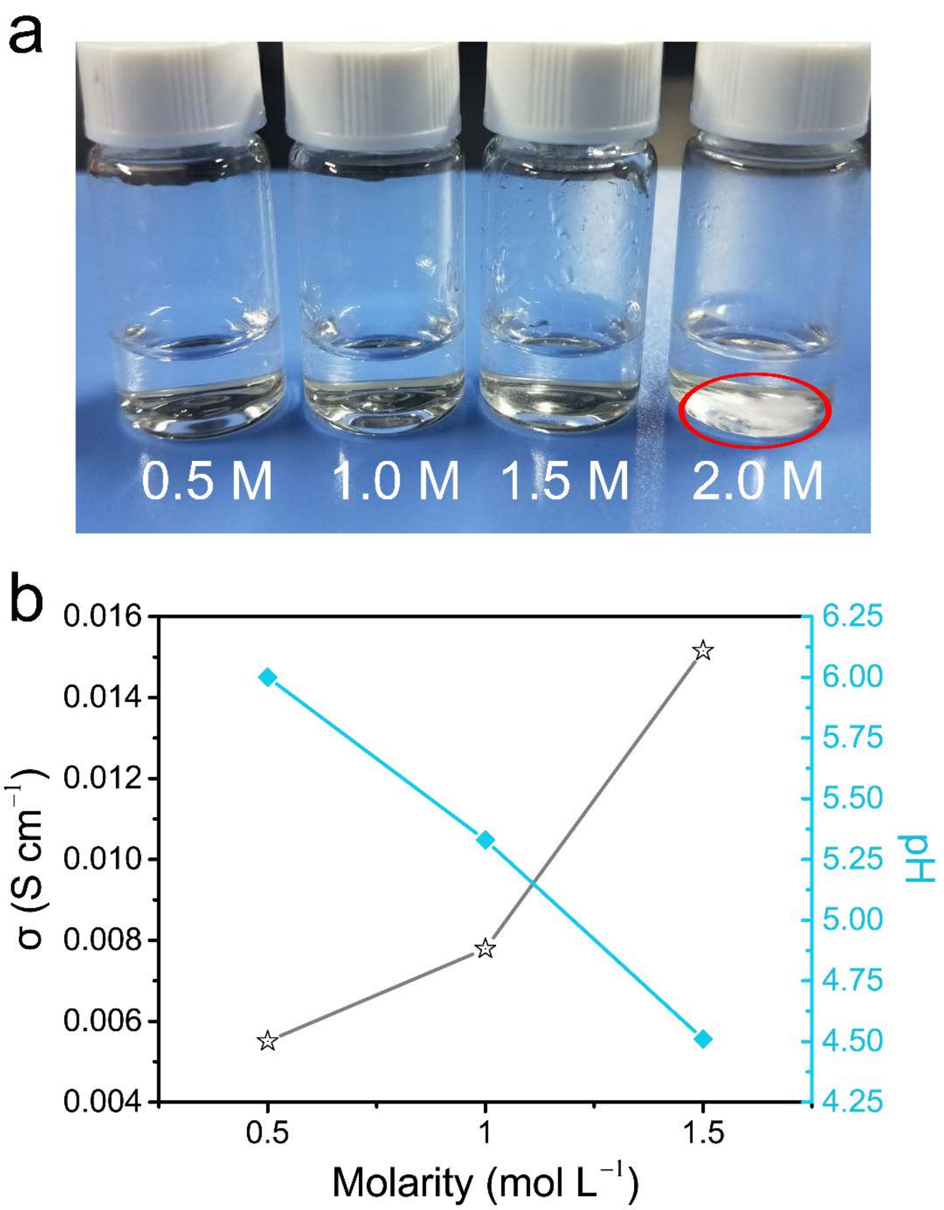

Supplementary Fig. 14 Screening of the control group. (a) Images of $Zn(TFSI)_2$ aqueous electrolytes (after resting for 24 hours). **(b)** Variation of pH and conductivity of $Zn(TFSI)_2$ aqueous electrolytes with different concentrations.

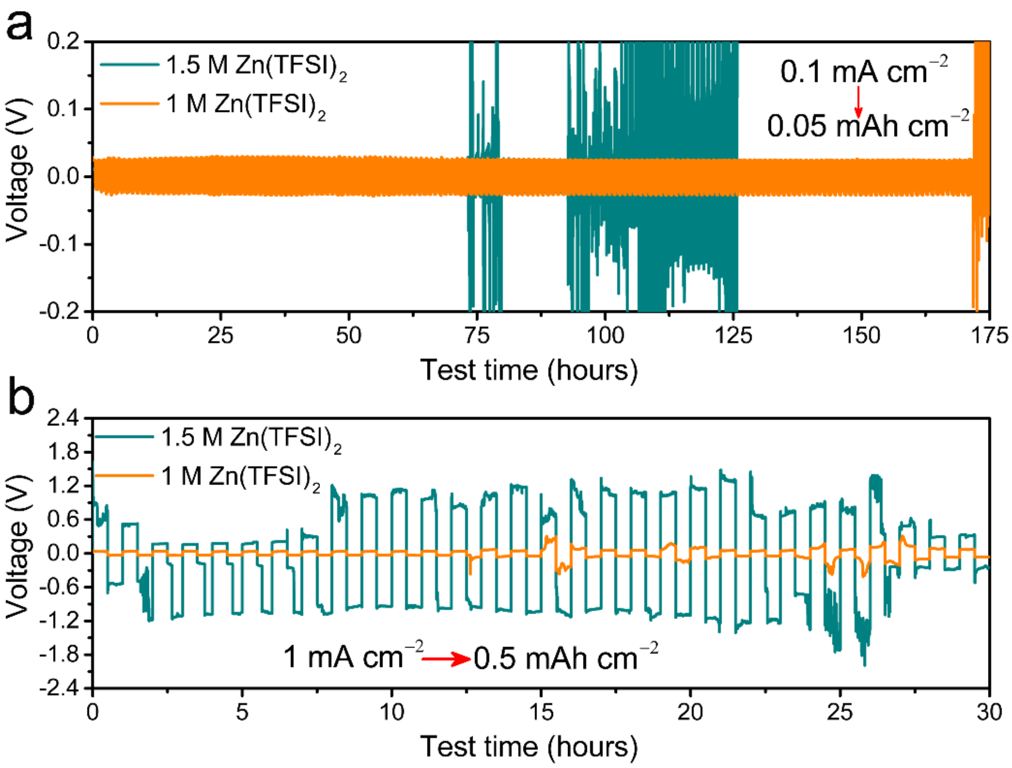

Supplementary Fig. 15 Screening of the control group. Voltage responses of Zn/Zn symmetric cells in 1.5 M and 1 M $Zn(TFSI)_2$ aqueous electrolytes at **(a)** 0.1 mA cm^{-2} (0.05 mAh cm^{-2} for each half cycle) and **(b)** 1 mA cm^{-2} (0.5 mAh cm^{-2} for each half cycle), respectively. 1 M $Zn(TFSI)_2$ exhibits extended cycle life and lower polarization at both low and high rates compared with 1.5 M $Zn(TFSI)_2$.

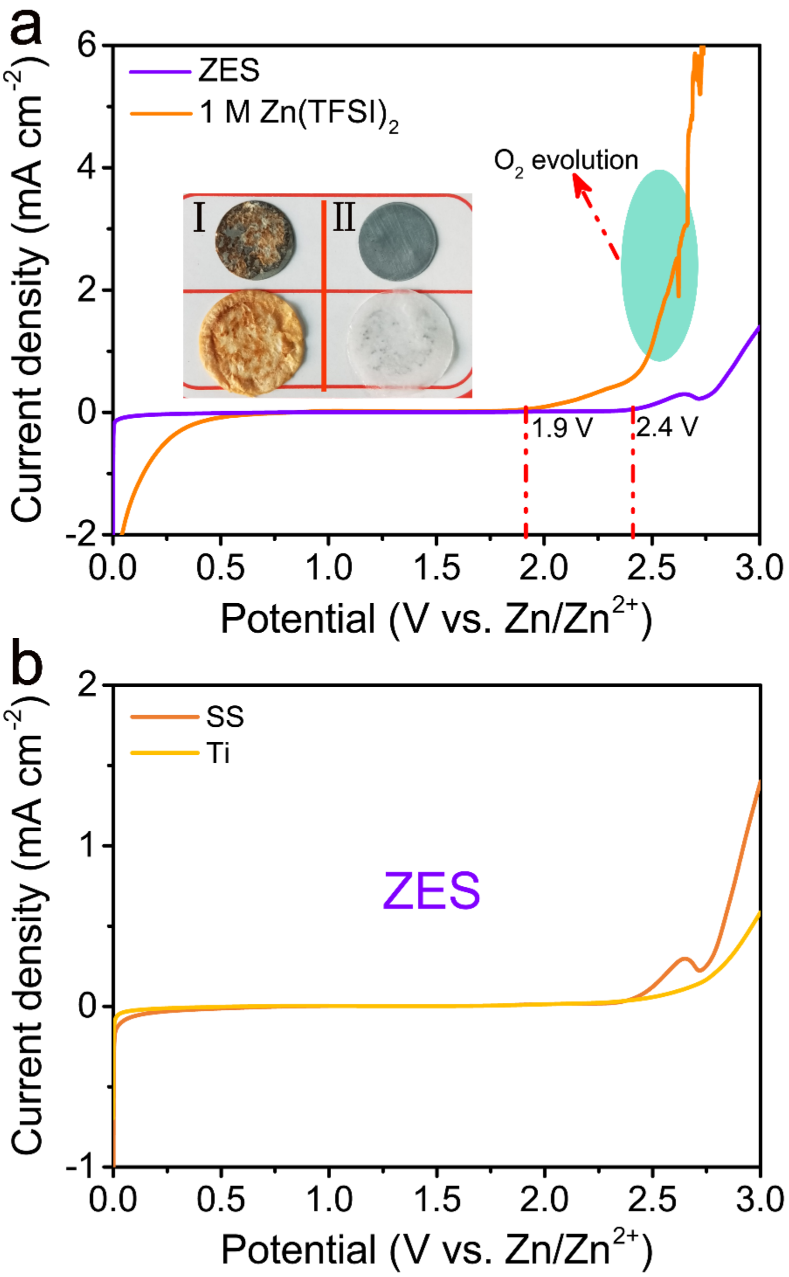

Supplementary Fig. 16 Electrochemical stability of various electrolytes. (a) Comparison of linear sweep voltammetry of ZES and 1 M $Zn(TFSI)_2$ in Zn/SS cells at the scanning rate of 1 mV/s. The working and counter electrodes are SS and Zn, respectively. Insets: the optical photographs of Zn anodes and separators after testing in **(I)** ZES and **(II)** 1 M $Zn(TFSI)_2$. **(b)** Linear sweep voltammetry of ZES at the scanning rate of 1 mV s^{-1} with SS and Ti as working electrodes, respectively.

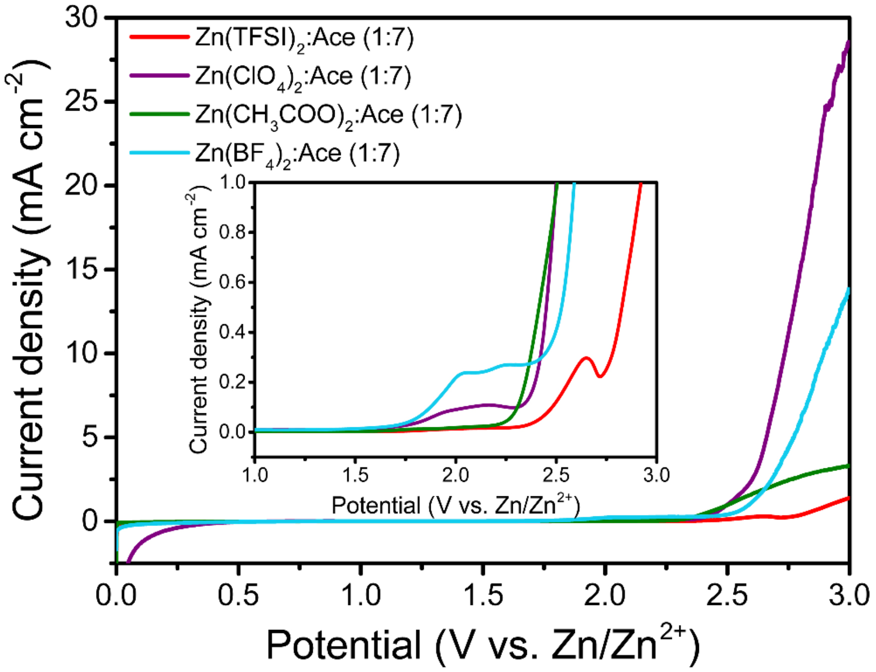

Supplementary Fig. 17 Comparison of linear sweep voltammetry of different DESs. The scanning rate is 1 mV s^{-1}, and the working electrode is SS.

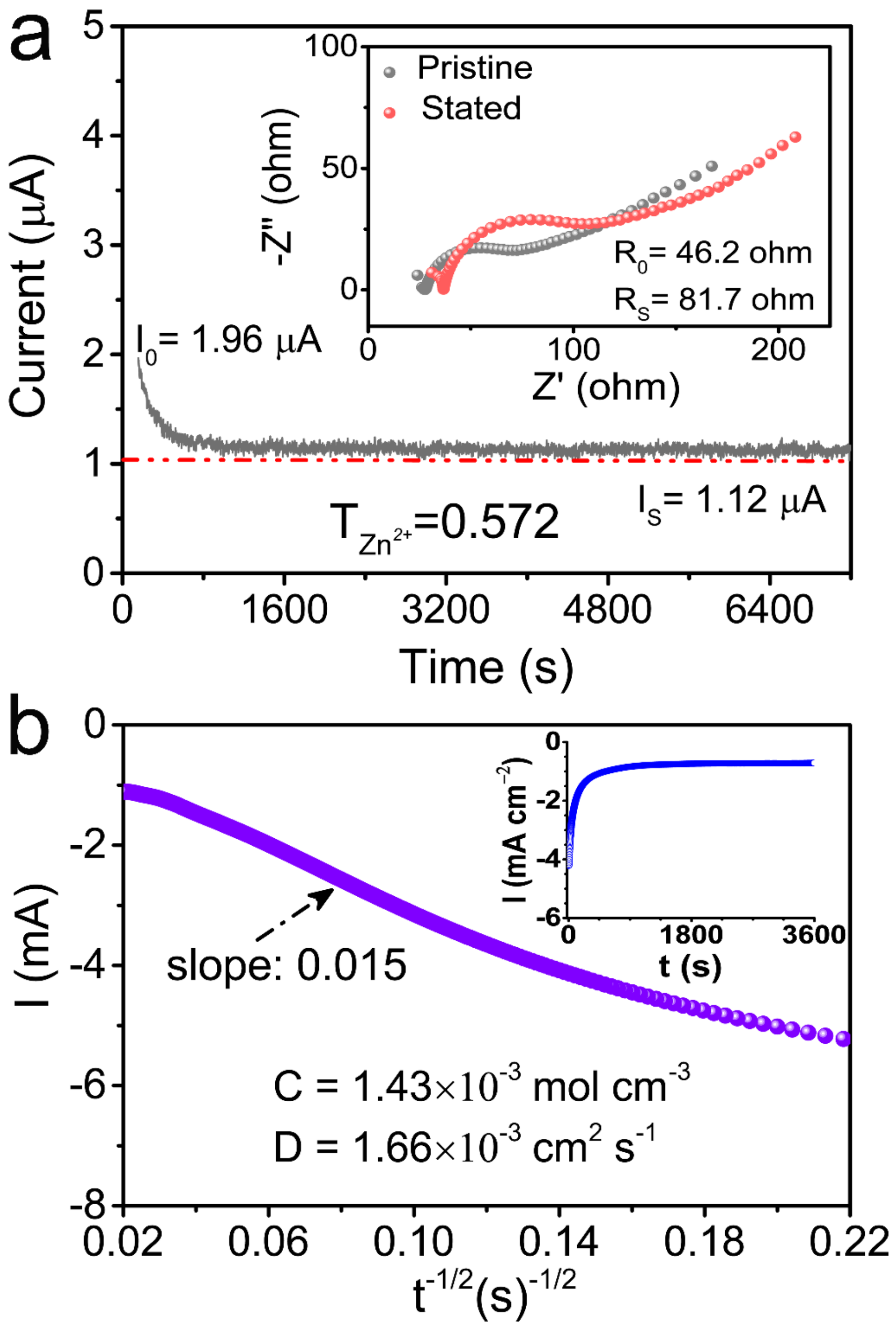

Supplementary Fig. 18 Ion-transport properties of the ZES. **(a)** Current-time curves following DC polarization of ZES at 0.005 V. Inset shows AC impedance spectra. **(b)** Plot of current vs. time$^{-1/2}$ for ZES. Both working and counter electrodes are Zn. Potential Step: –0.2 V (*vs.* Zn/Zn^{2+}).

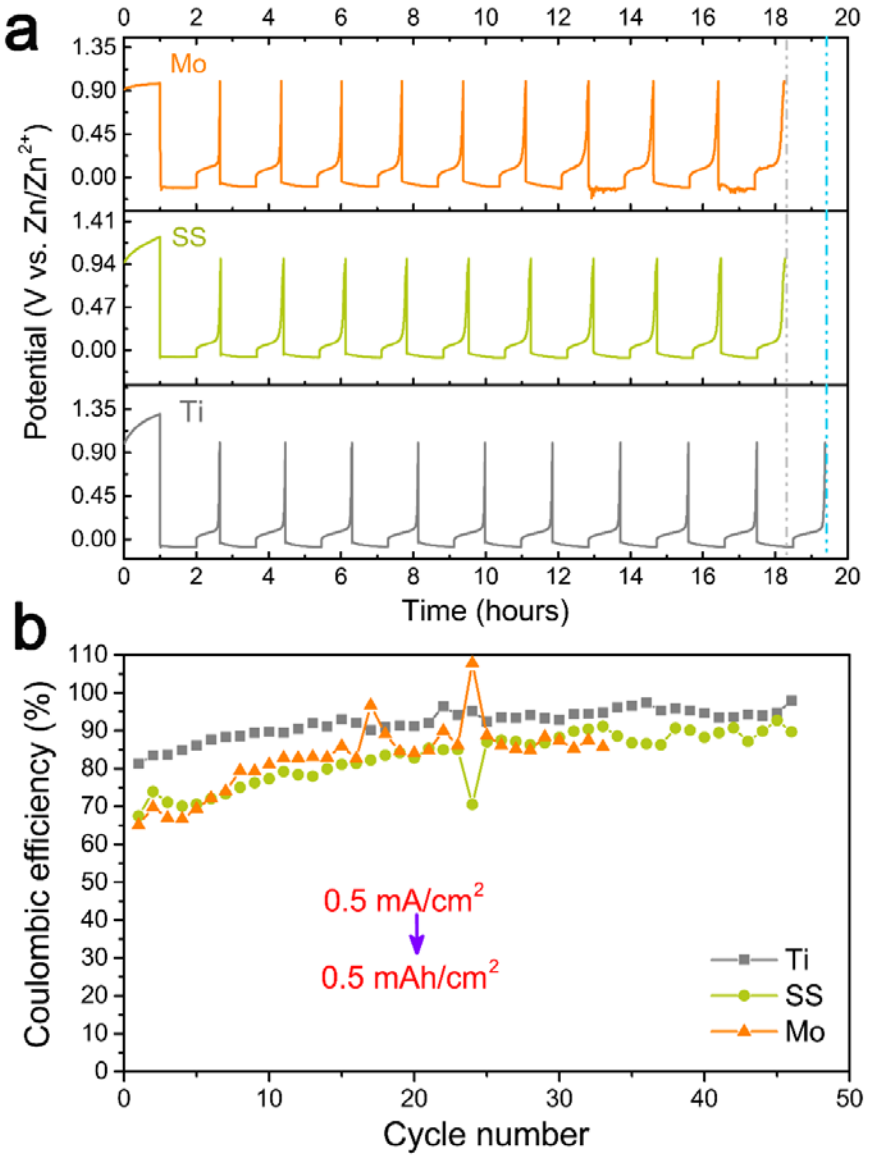

Supplementary Fig. 19 The optimization of working electrodes. (a) Voltage profiles and **(b)** corresponding CE of Zn plating/stripping on Ti, SS and Mo working electrodes (with same treatment condition), respectively, at 0.5 mA cm^{-2} (0.5 mAh cm^{-2}) in ZES. It is apparent that the cell exhibits the highest CE of Zn plating/stripping when Ti using as the working electrode.

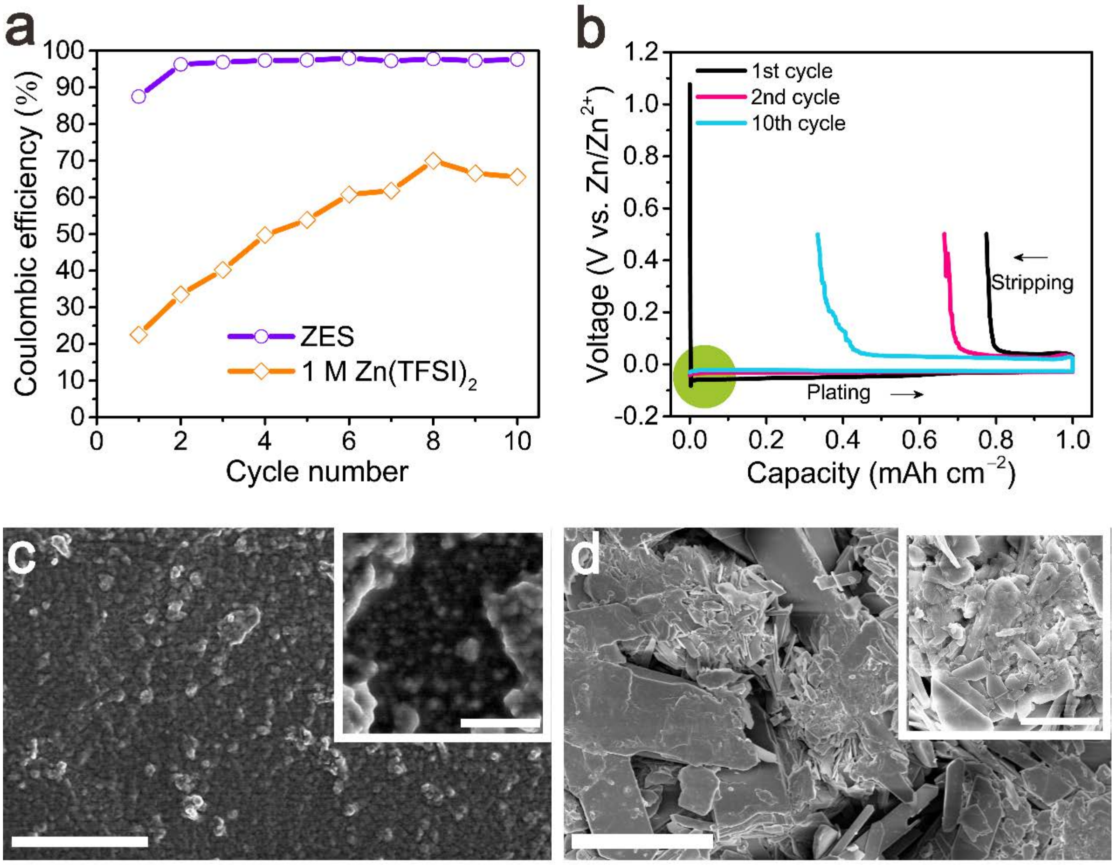

Supplementary Fig. 20 CE test results and SEM images of Zn deposits after tests. (a) CE of Zn plating/stripping vs. cycle number plot for Zn/Ti cells cycled at 0.5 mA cm^{-2} and a deposition capacity of 1 mAh cm^{-2}. **(b)** Galvanostatic Zn plating/stripping at 0.5 mA cm^{-2} in 1 M $Zn(TFSI)_2$. The working and counter electrodes are Ti and Zn, respectively. SEM images of Zn deposits on Ti after 10 cycles of galvanostatic Zn plating/stripping at a 0.5 mA cm^{-2} (1 mAh cm^{-2}) in **(c)** ZES and **(d)** 1 M $Zn(TFSI)_2$. The working and counter electrodes are Ti and Zn, respectively. Scale bar: 50 μm for **c**, **d**; 5 μm for inset in **c**; 10 μm for inset in **d**.

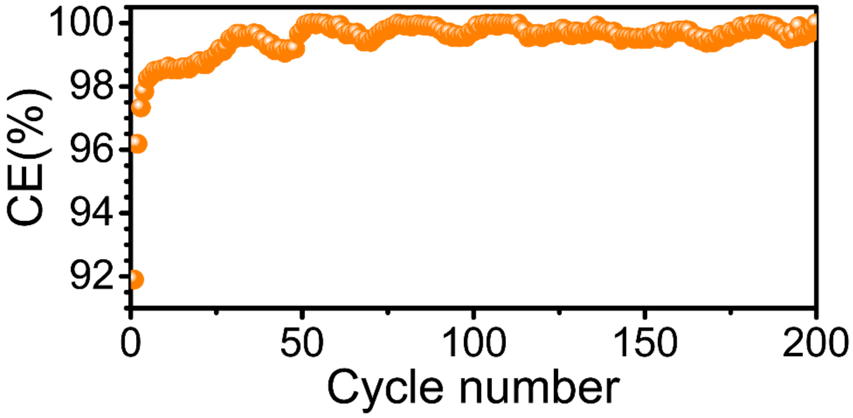

Supplementary Fig. 21 The variation of Zn plating/stripping CE with cycling based on CV.

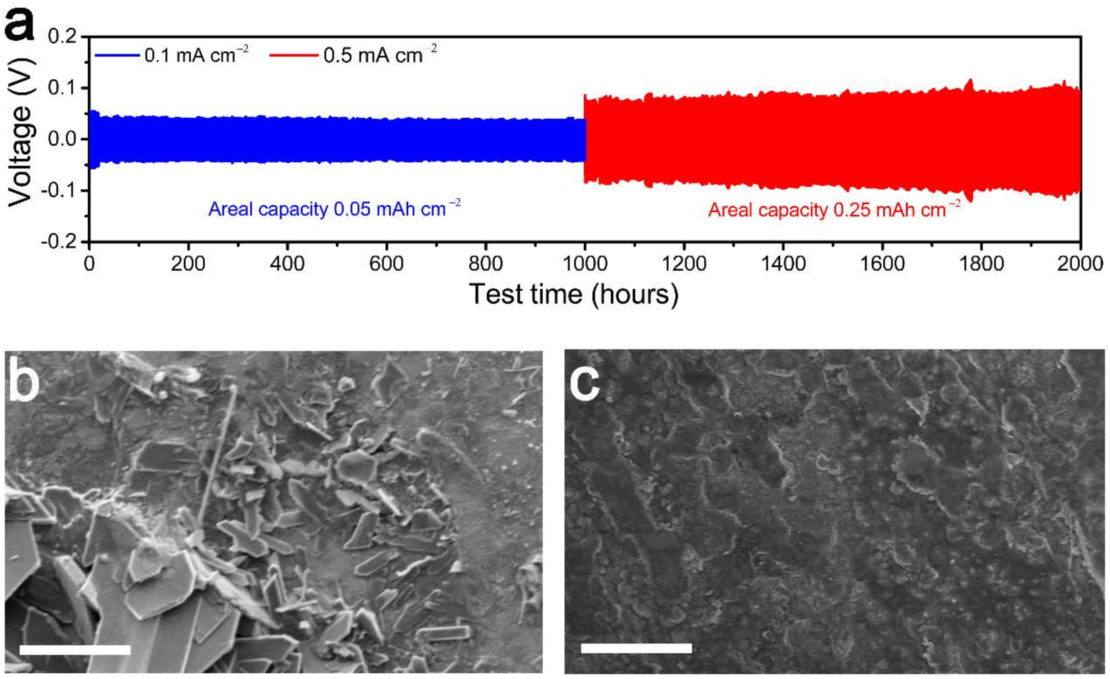

Supplementary Fig. 22 The Zn plating/stripping behavior and SEM images of Zn deposits. **(a)** Voltage responses of the Zn/Zn symmetric cell under repeated polarization in ZES at 0.1 mA cm^{-2} (before the 1,000th cycle) and 0.5 mA cm^{-2} (after the 1,000th cycle). SEM images of the cycled Zn after **(b)** 180 cycles in 1 M $Zn(TFSI)_2$ and **(c)** 2,000 cycles in ZES at a rate of 0.1 mA cm^{-2} (0.05 mAh cm^{-2}), respectively. Scale bar: 30 μm for **b**, **c**.

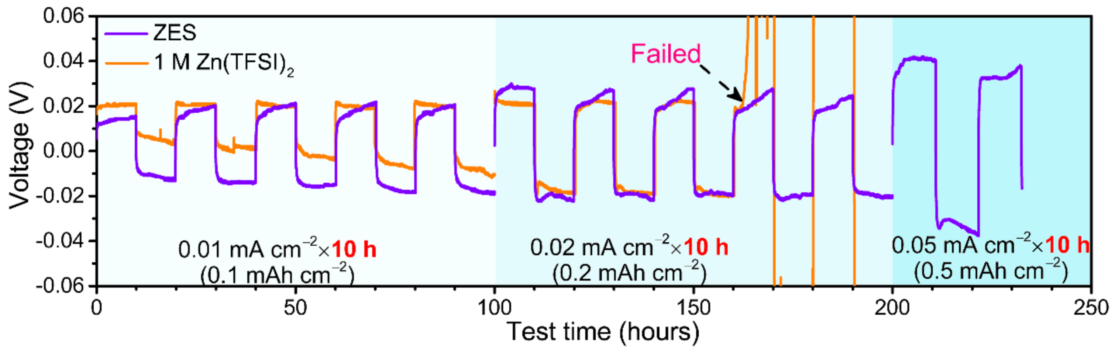

Supplementary Fig. 23 Comparison of Zn plating/stripping in different electrolytes. Voltage responses of Zn/Zn symmetric cells in ZES and 1 M $Zn(TFSI)_2$ at low rates of 0.01−0.05 mA cm^{-2} (each half cycle lasts for 10 hours). The cell with ZES performed satisfactorily even at a rate of 0.05 mA cm^{-2} (0.5 mAh cm^{-2}), while that with 1 M $Zn(TFSI)_2$ failed at 0.02 mA cm^{-2} (0.2 mAh cm^{-2}).

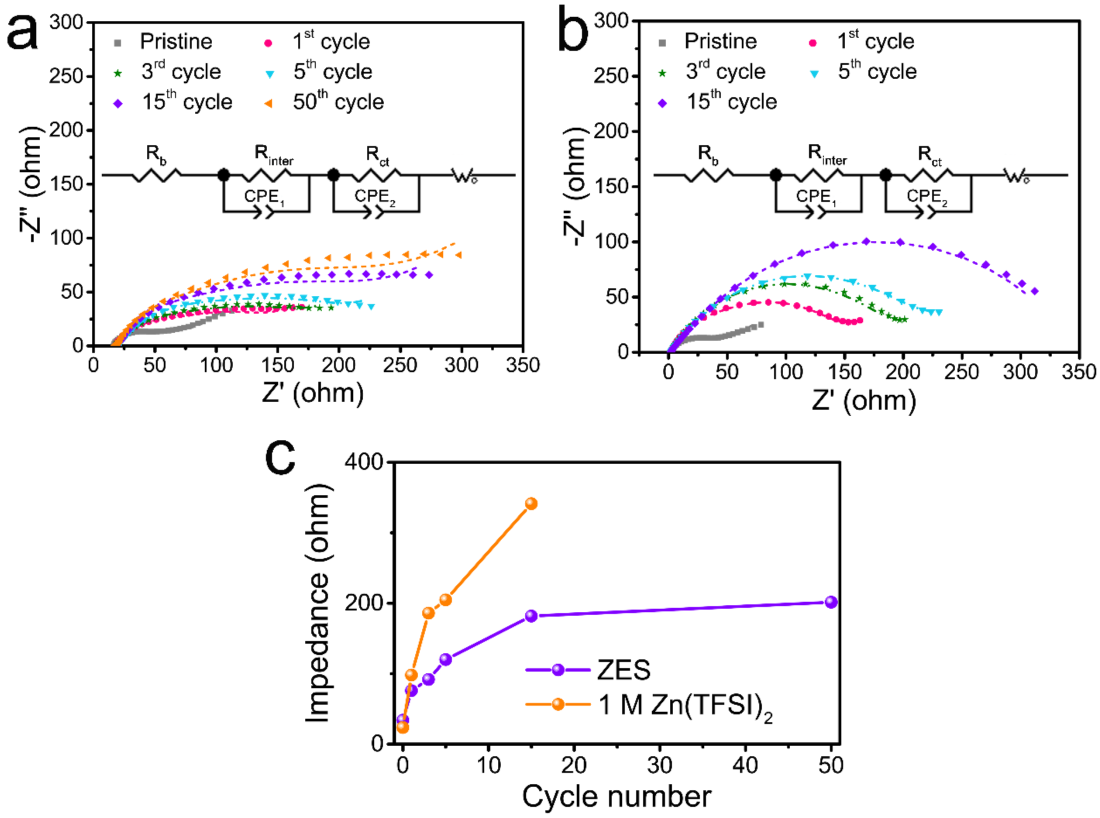

Supplementary Fig. 24 The Zn-electrolyte interface stability in different electrolytes. EIS data measured with Zn/Zn symmetric cells in **(a)** ZES and **(b)** 1 M $Zn(TFSI)_2$ at different galvanostatic cycles at 0.1 mA cm^{-2}. Scatter plots and dashed lines denote experimental spectra and fitting curves of impedance, respectively. **(c)** The evolution of the charge-transfer resistance in different electrolytes. Insets in **(a)** and **(b)** exhibit the equivalent circuit model of EIS. R_b stands for the electrolyte bulk resistance, R_{inter} and CPE_1 are the interface resistance and its related double-layer capacitance, which correspond to the semicircle at high frequencies, R_{ct} and CPE_2 represent the charge transfer resistance and its related double-layer capacitance, which correspond to the semicircle at medium frequencies, and W_O represents the Warburg impedance related to the diffusion of Zn-ions, which is indicated at low frequencies[25].

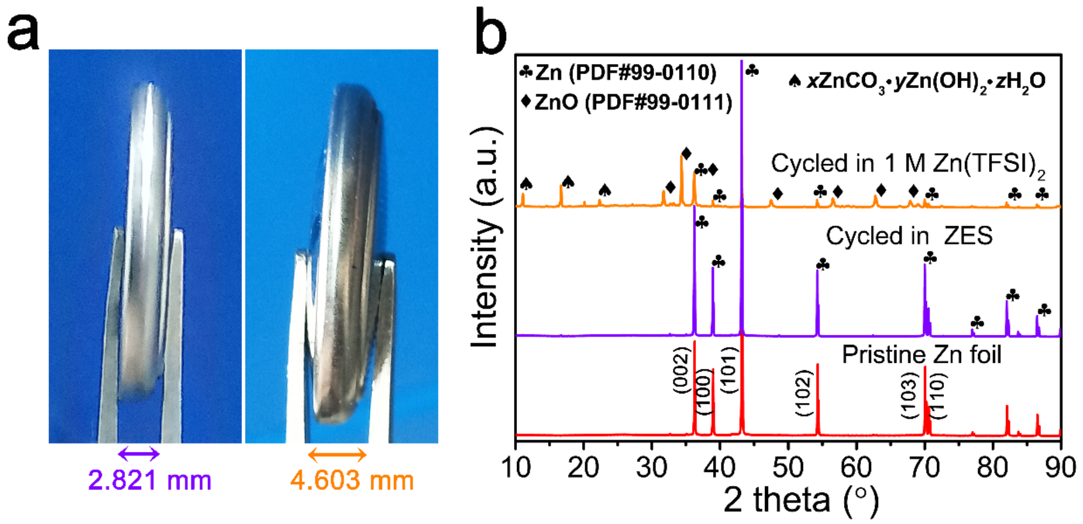

Supplementary Fig. 25 Chemical composition analysis of the cycled Zn-electrolyte interface. (a) Optical images of Zn/Zn cells in ZES (left) and 1 M Zn(TFSI)$_2$ (right) after plating/stripping tests for 30 cycles at 1 mA cm^{-2}. **(b)** XRD patterns of Zn anodes after plating/stripping tests (0.1 mA cm^{-2}) for 2,000 cycles in ZES and 180 cycles in 1 M Zn(TFSI)$_2$, respectively.

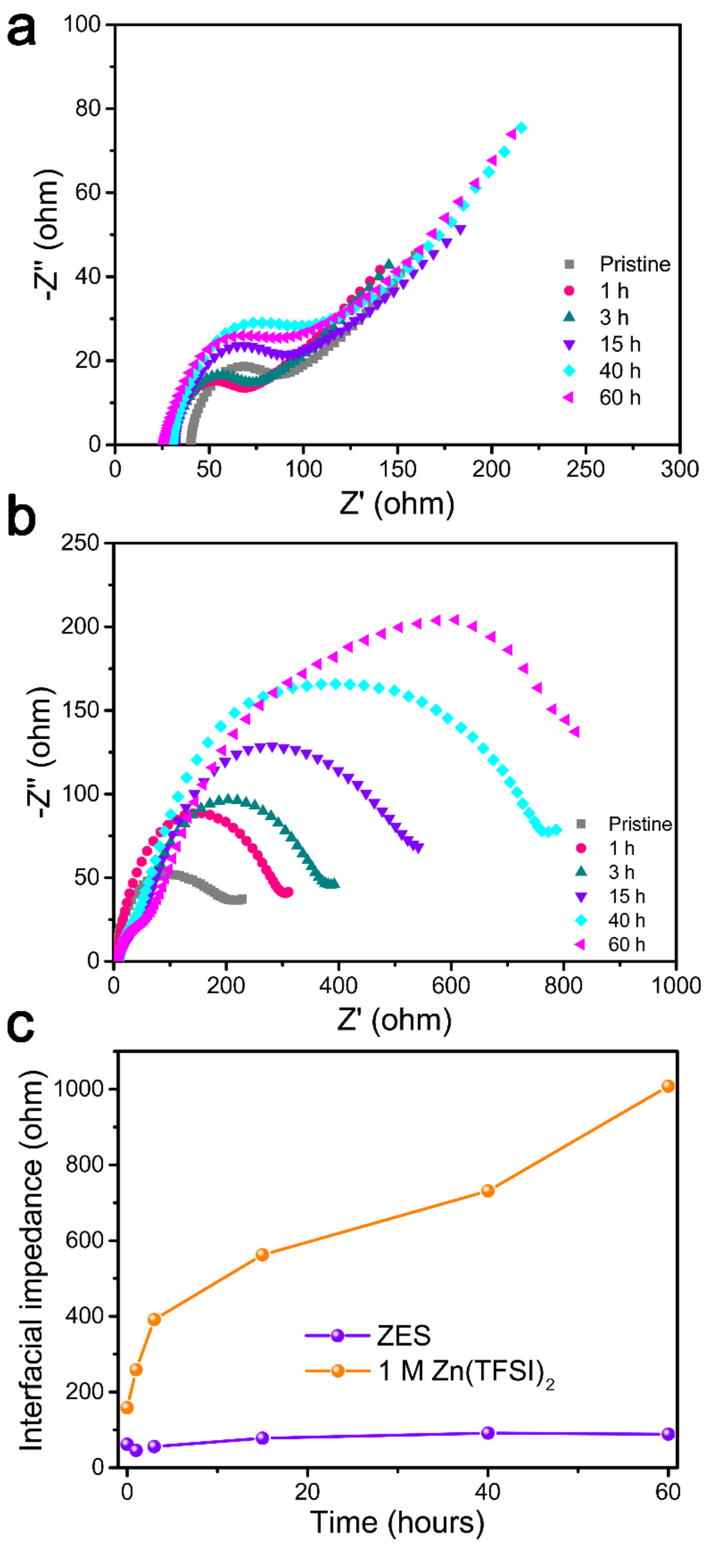

Supplementary Fig. 26 The chemical stability of the metallic Zn in ZES. EIS plots of Zn/Zn cells in **(a)** ZES and **(b)** 1 M $Zn(TFSI)_2$ under open circuit conditions as a function of standing time. **(c)** Time evolution of impedance. Impedance in the case of ZES initially increases, and reaches a stable value of about 100 ohm after 40 h.

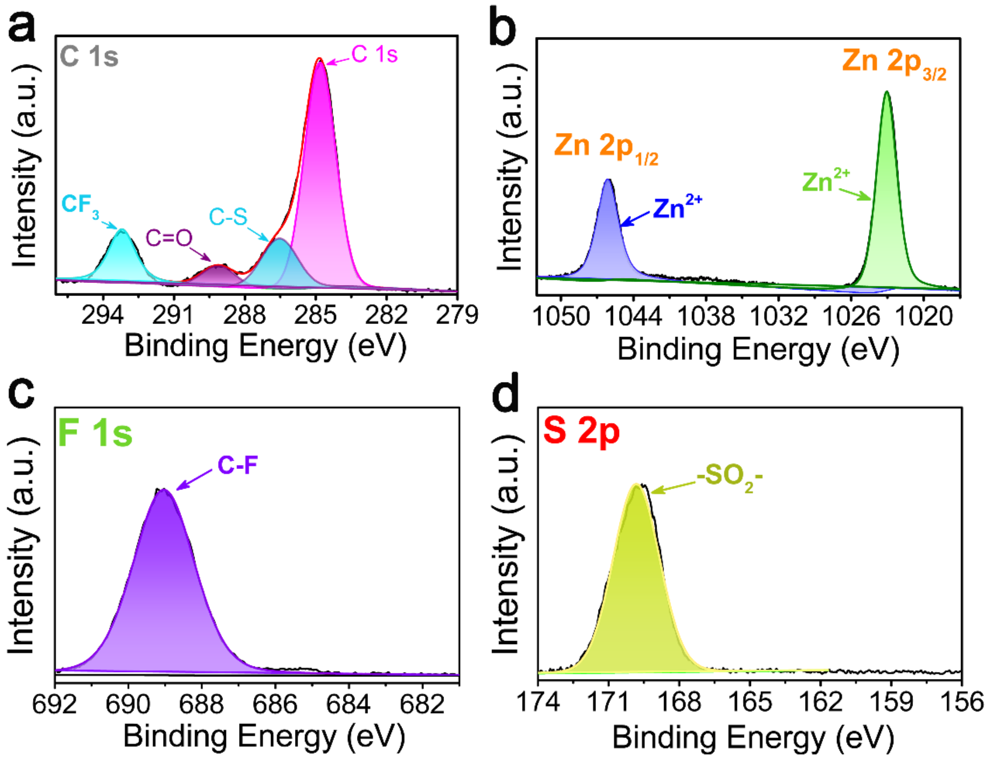

Supplementary Fig. 27 The chemical composition analysis of Zn(TFSI)$_2$. (a) C 1s, **(b)** Zn 2p, **(c)** F 1s, and **(d)** S 2p XPS spectra of the pure Zn(TFSI)$_2$.

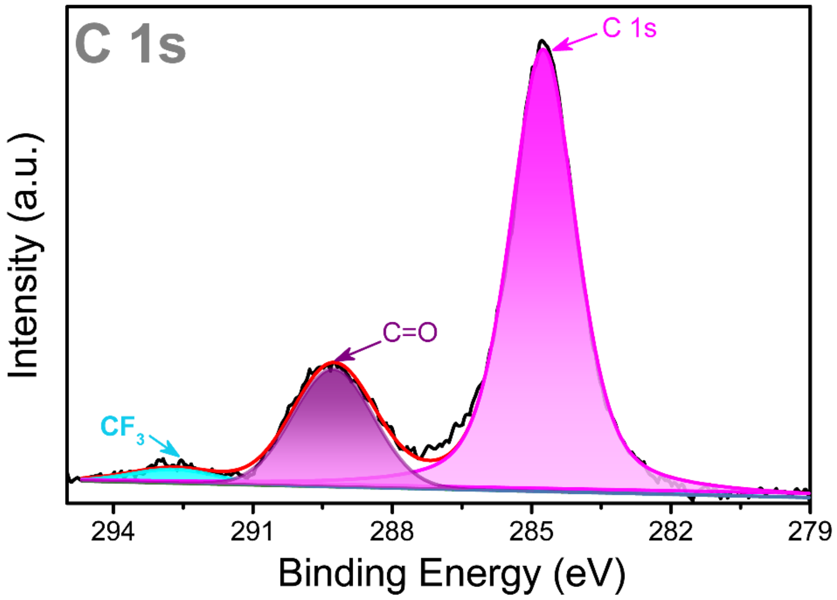

Supplementary Fig. 28 XPS spectrum of the C 1s region of Zn surface after 20 cycles in a Zn/Zn cell under galvanostatic condition (0.5 mA cm^{-2} with a capacity of 1 mAh cm^{-2} for each half cycle).

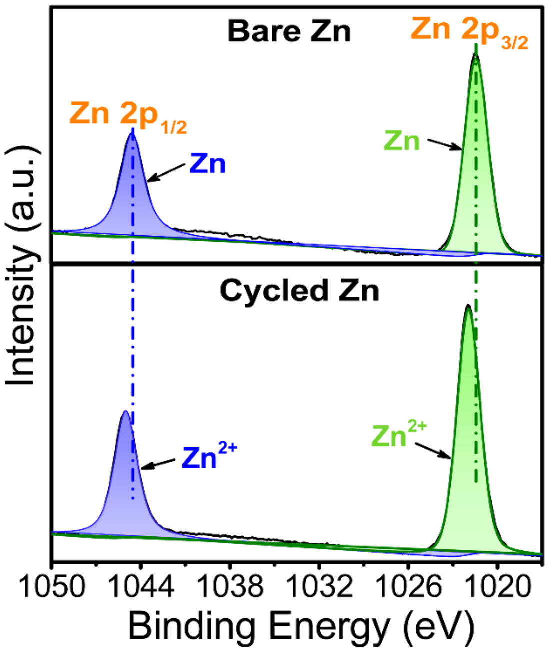

Supplementary Fig. 29 The composition of the Zn-compatible SEI layer. XPS spectra of the Zn 2p region of Zn surface in bare Zn (upper) and cycled Zn after 20-cycled charge/discharge testing (lower) (under galvanostatic condition, 0.5 mA cm^{-2} with a capacity of 1 mAh cm^{-2} for each half cycle). The peaks at 1021.52 and 1044.58 eV correspond to metallic Zn[2].

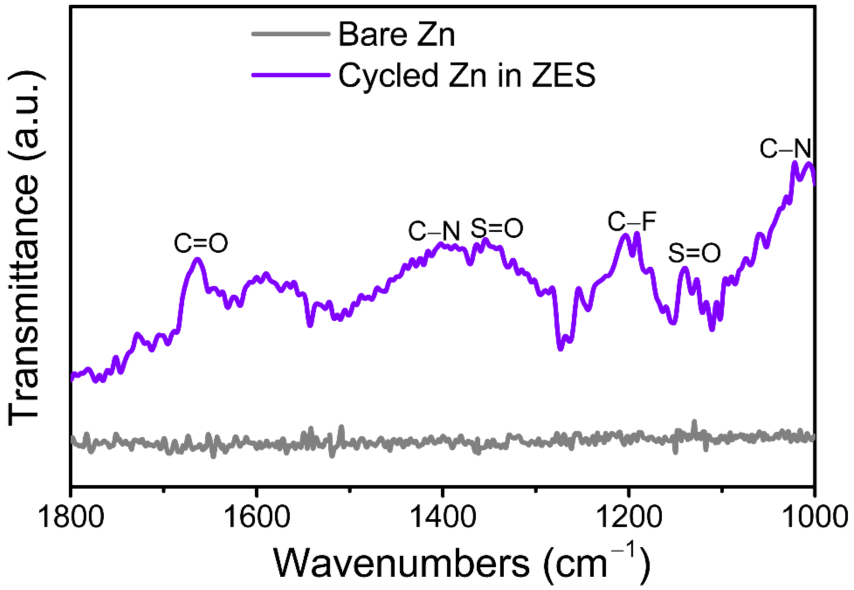

Supplementary Fig. 30 FTIR peaks of the SEI on the surface of 20-cycled charge/discharge Zn anode in ZES (0.5 mA cm^{-2} with a capacity of 1 mAh cm^{-2} for each half cycle).

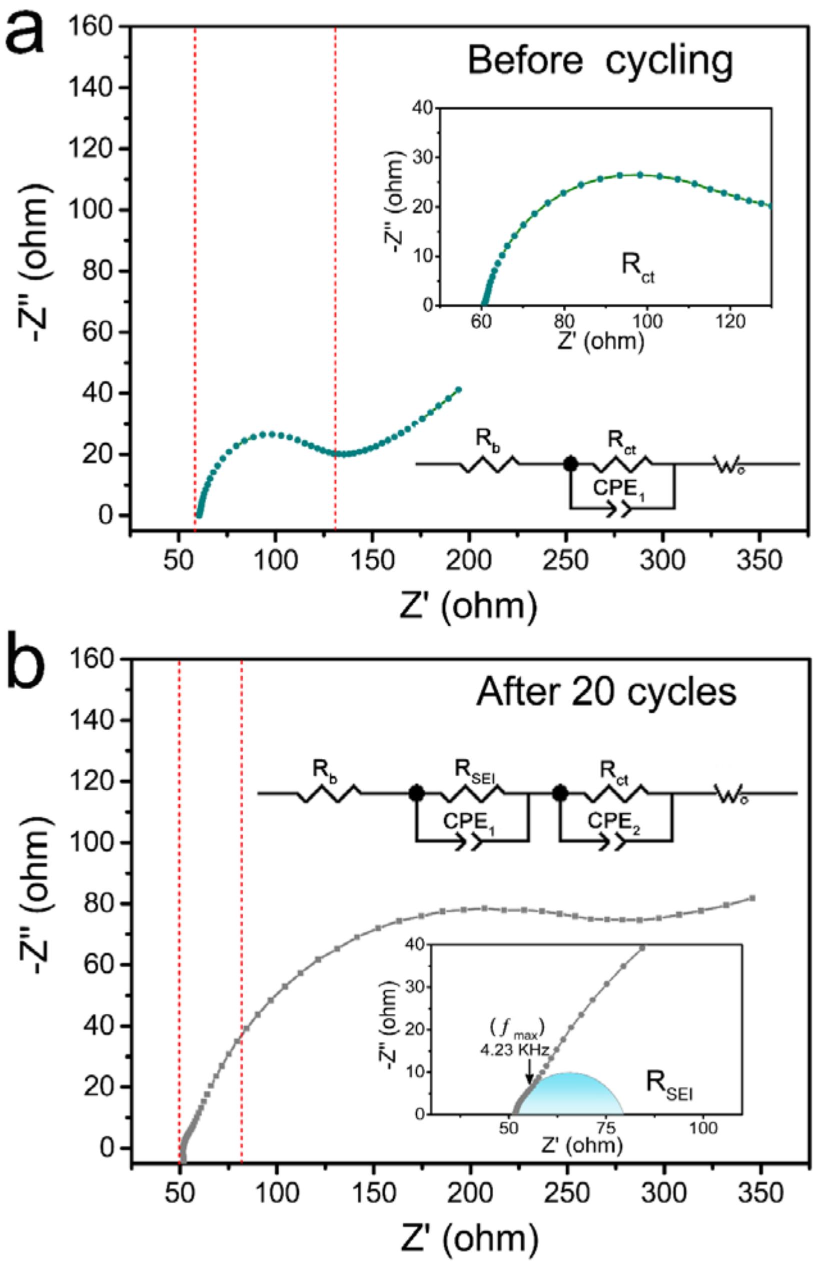

Supplementary Fig. 31 EIS spectra of the Zn/Zn cell (a) before cycling and **(b)** after forming stable SEI (under galvanostatic condition for 20 cycles, 0.5 mA cm^{-2} with a capacity of 1 mAh cm^{-2}) in ZES. Insets: enlarged images in the dotted boxes of panel and data fitting of impedance spectra. R_b stands for the electrolyte bulk resistance, R_{SEI} and CPE_1 are the interface resistance and its related double-layer capacitance, which correspond to the semicircle at high frequencies, R_{ct} and CPE_2 represent the charge transfer resistance and its related double-layer capacitance, which correspond to the semicircle at medium frequencies, and W_O represents the Warburg impedance related to the diffusion of Zn-ions, which is indicated at low frequencies.

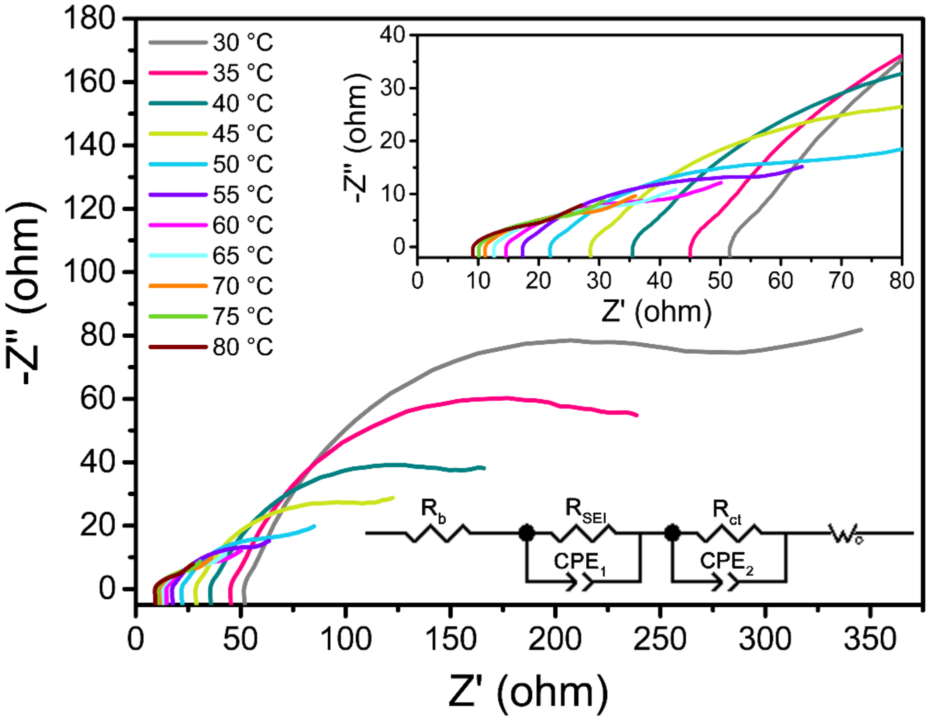

Supplementary Fig. 32 EIS spectra of the Zn/Zn cell after forming stable SEI at different temperatures in ZES (under galvanostatic condition for 20 cycles, 0.5 mA cm^{-2} with a capacity of 1 mAh cm^{-2} for each half cycle). Insets: enlarged image and data fitting of impedance spectra.

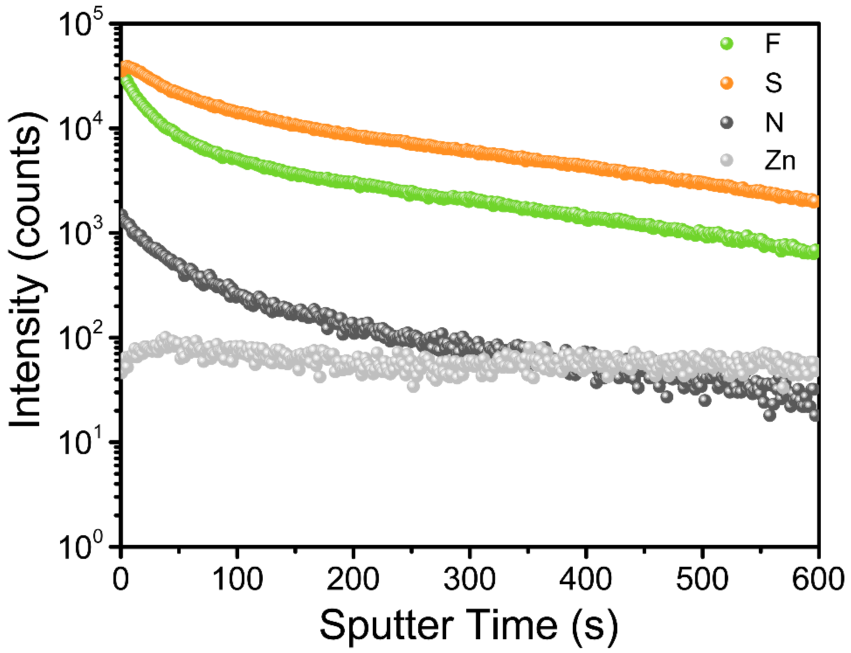

Supplementary Fig. 33 TOF-SIMS depth-profiles of Zn, N, F, and S elements of SEI-coated Zn anode.

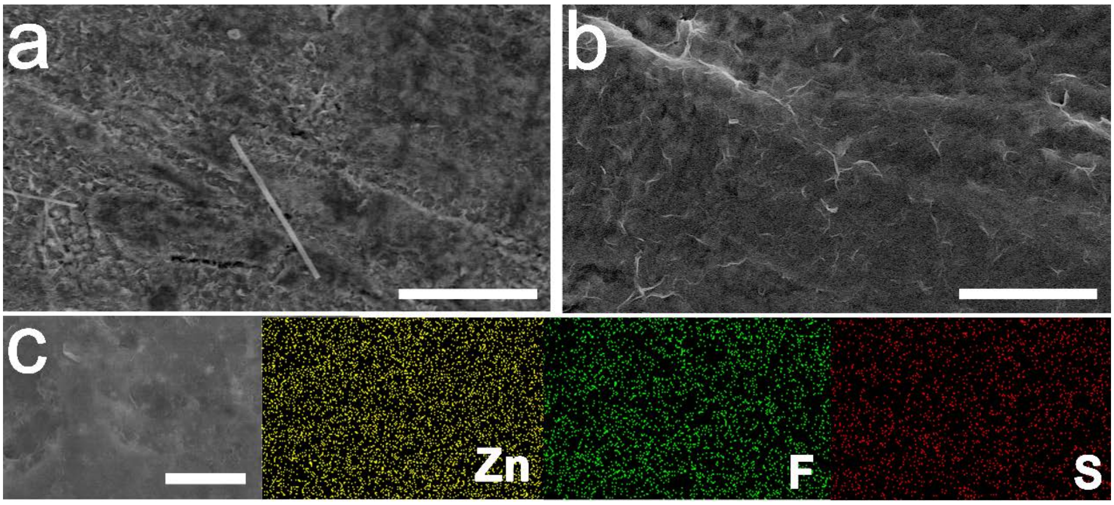

Supplementary Fig. 34 Surface characteristics of the cycled Zn in ZES. (a, b) SEM images and **(c)** EDS mapping results of the 20-cycled Zn (under galvanostatic condition for 20 cycles, 0.5 mA cm^{-2} with a capacity of 1 mAh cm^{-2} for each half cycle). Scale bar: 10 μm for **a**, 3 μm for **b** and 2 μm for **c**.

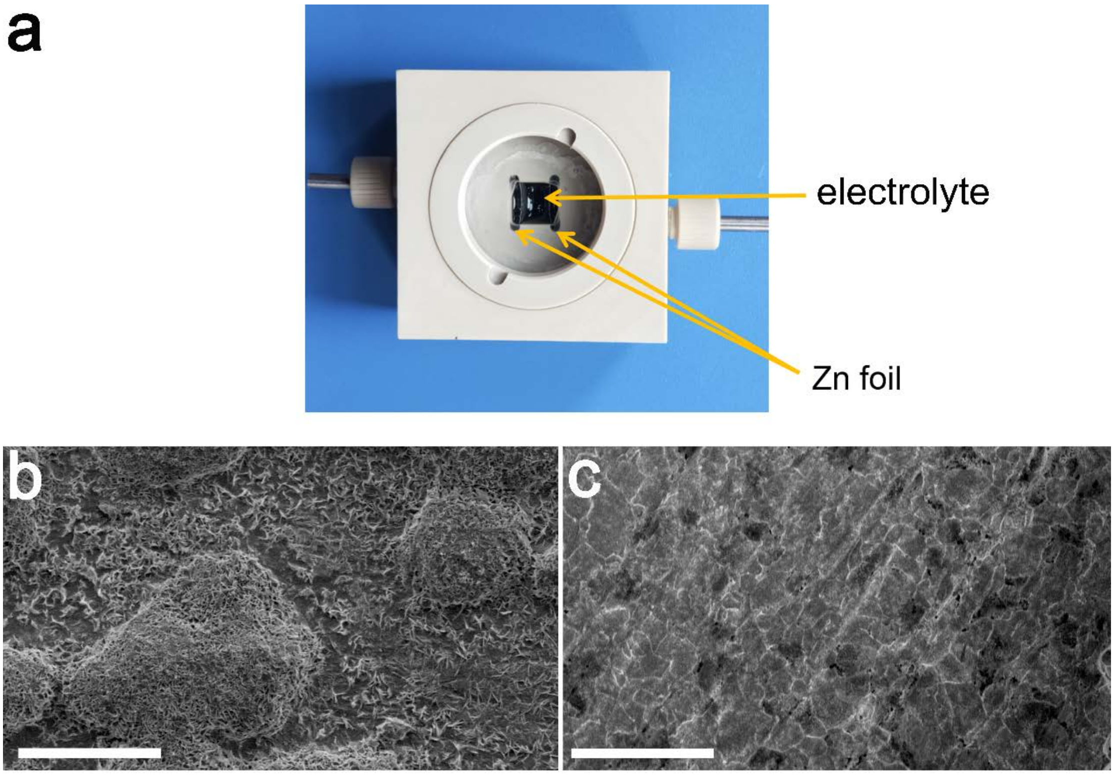

Supplementary Fig. 35 Effect of ZES and as-obtained SEI layer on Zn deposition. (a) The home-made cell for operando optical microscopy adopted to investigate in-situ Zn plating behavior. SEM images of Zn deposits in **(b)** 1 M $Zn(TFSI)_2$ and **(c)** ZES with a areal capacity 5 mAh cm^{-2} (10 mA cm^{-2}). Scale bar: 50 μm for **b**, **c**.

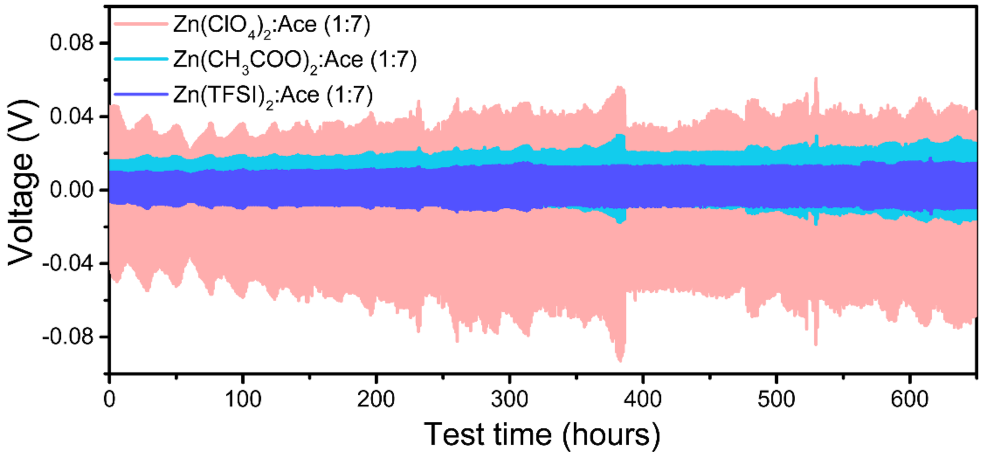

Supplementary Fig. 36 Comparison of DES electrolytes. Voltage responses of Zn/Zn symmetric cells in DES electrolytes formed by different Zn salts ($Zn(ClO_4)_2$, $Zn(CH_3COO)_2$ and $Zn(TFSI)_2$) at 0.01 mA cm^{-2} (0.5 h for each half cycle).

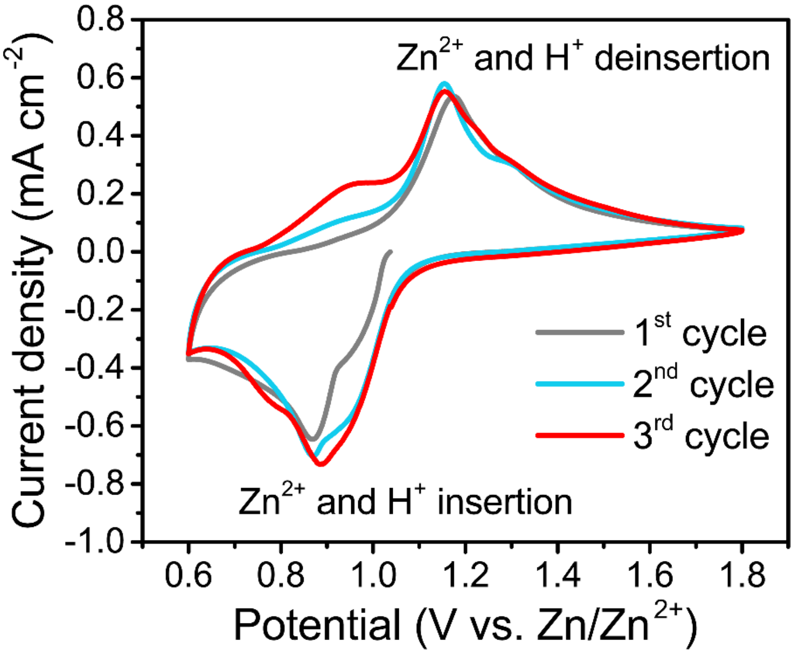

Supplementary Fig. 37 CV curves of the Zn/V_2O_5 cell using 1 M Zn(TFSI)$_2$ at a scan rate of 0.5 mV s^{-1}.

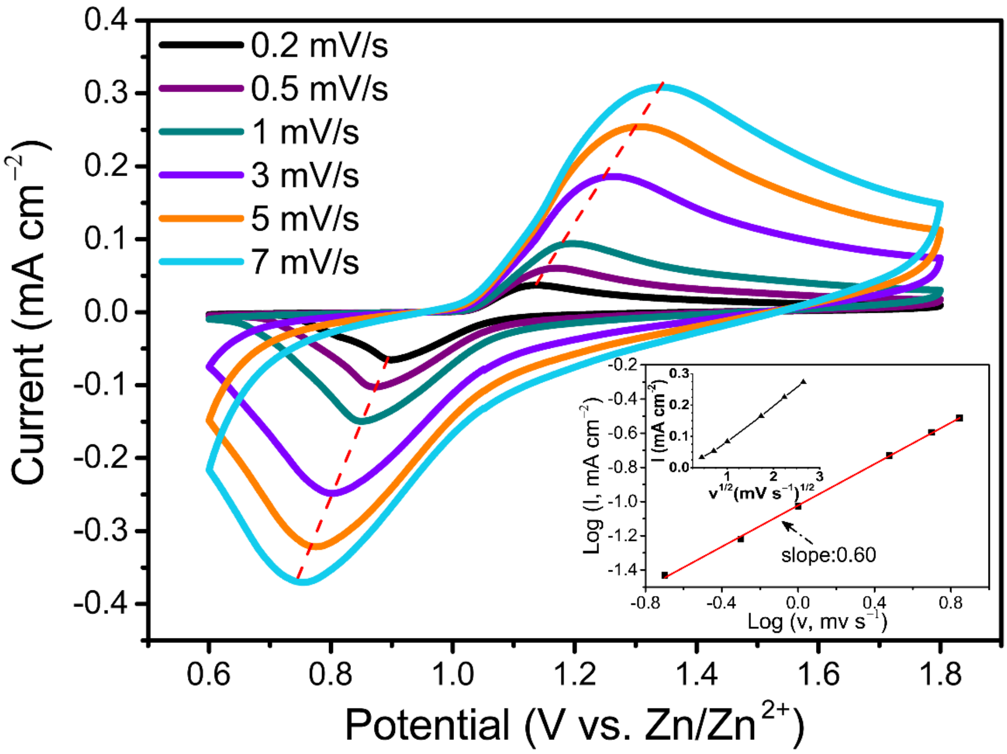

Supplementary Fig. 38 CV curves of the Zn/V_2O_5 cell with ZES as electrolyte (inset: the relationship between the peak current and scan rate in logarithmic format), confirming the diffusion-controlled redox reactions.

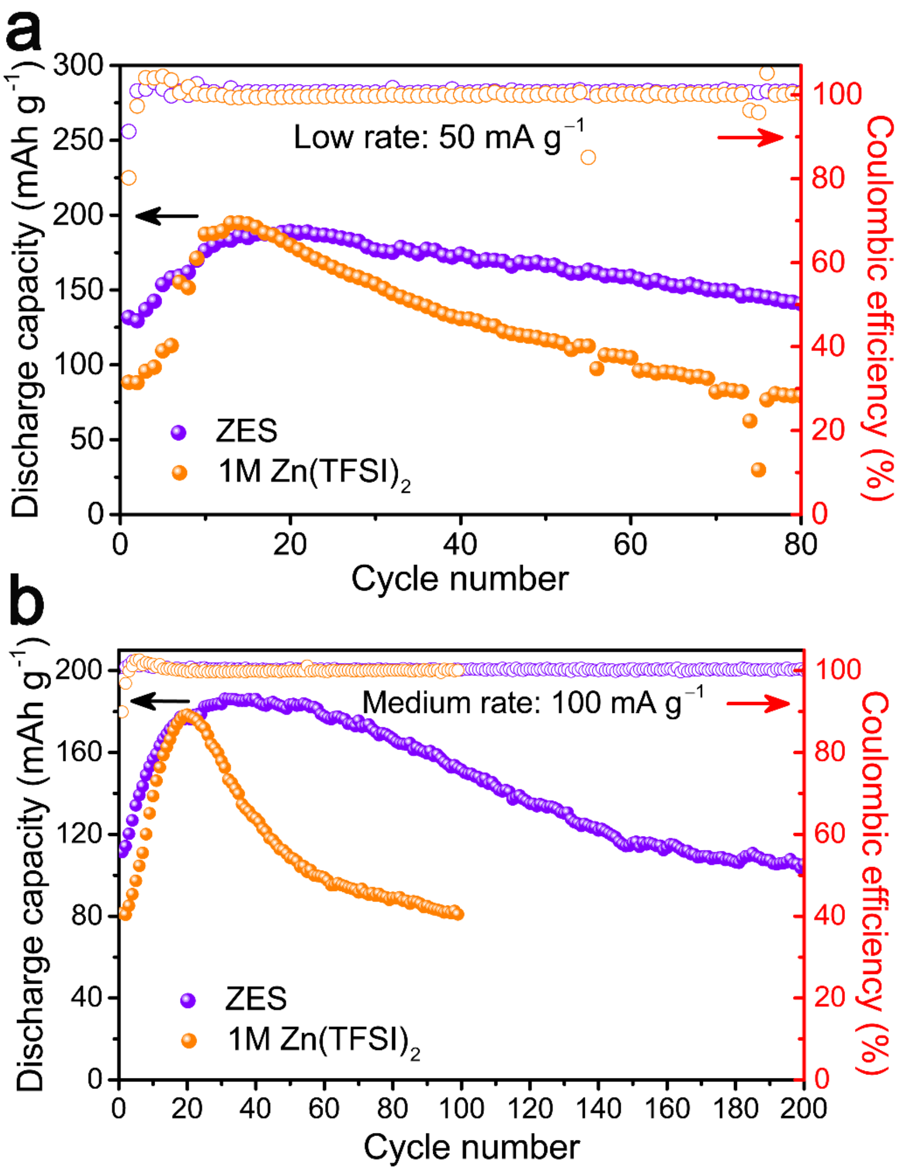

Supplementary Fig. 39 Electrochemical performance of the Zn/V_2O_5 cells. Cycling performance and CE of Zn/V_2O_5 cells in ZES and 1 M Zn(TFSI)$_2$ at **(a)** 50 mA g^{-1} and **(b)** 100 mA g^{-1}, respectively.

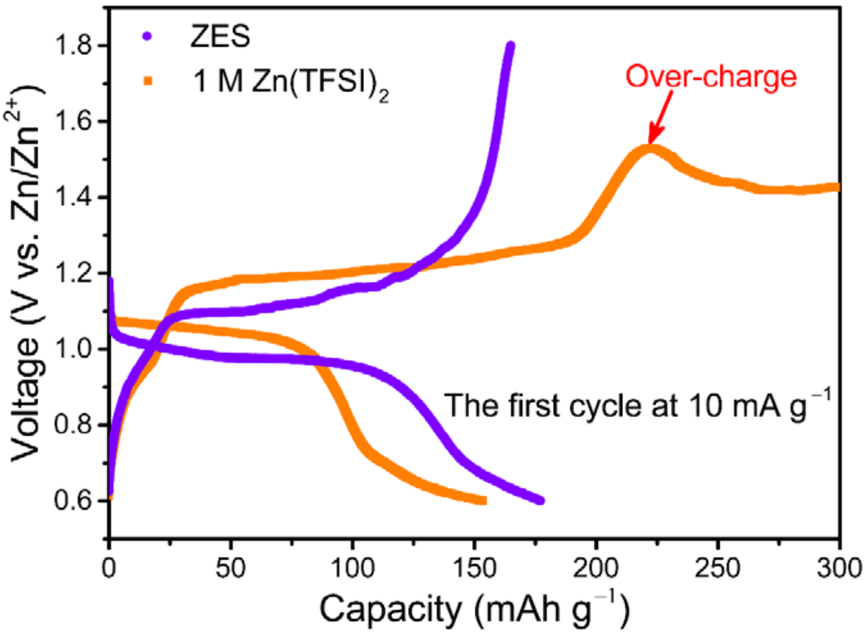

Supplementary Fig. 40 Galvanostatic charge/discharge curves of Zn/V_2O_5 cells at the first cycle.

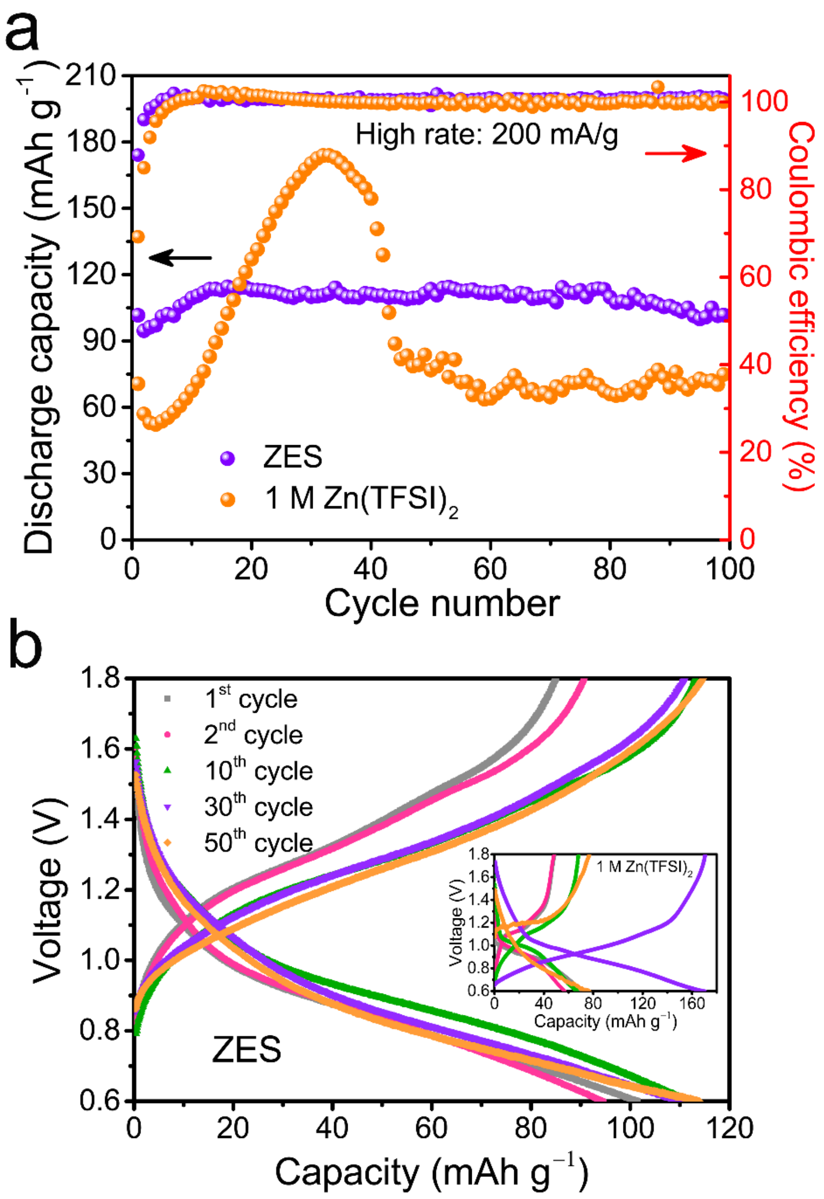

Supplementary Fig. 41 Electrochemical performance of the Zn/V_2O_5 cells. (a) Cycling performance and CE of Zn/V_2O_5 cells with ZES and 1 M Zn(TFSI)$_2$ at 200 mA g^{-1}, respectively. **(b)** The first 50 cycle curves of Zn/V_2O_5 cells using ZES and 1 M Zn(TFSI)$_2$ (inset), respectively.

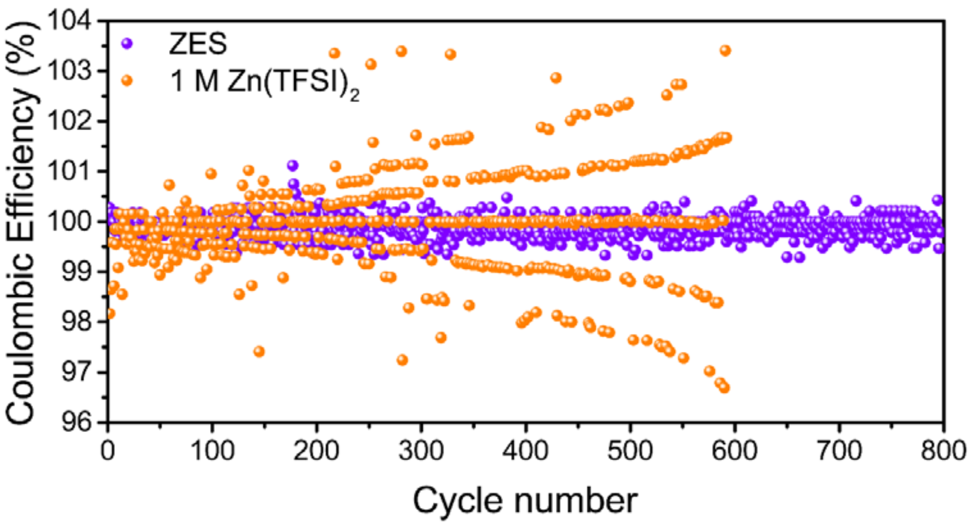

Supplementary Fig. 42 CE of Zn/V_2O_5 cells with ZES and 1 M Zn(TFSI)$_2$ electrolytes at 600 mA g^{-1}, respectively.

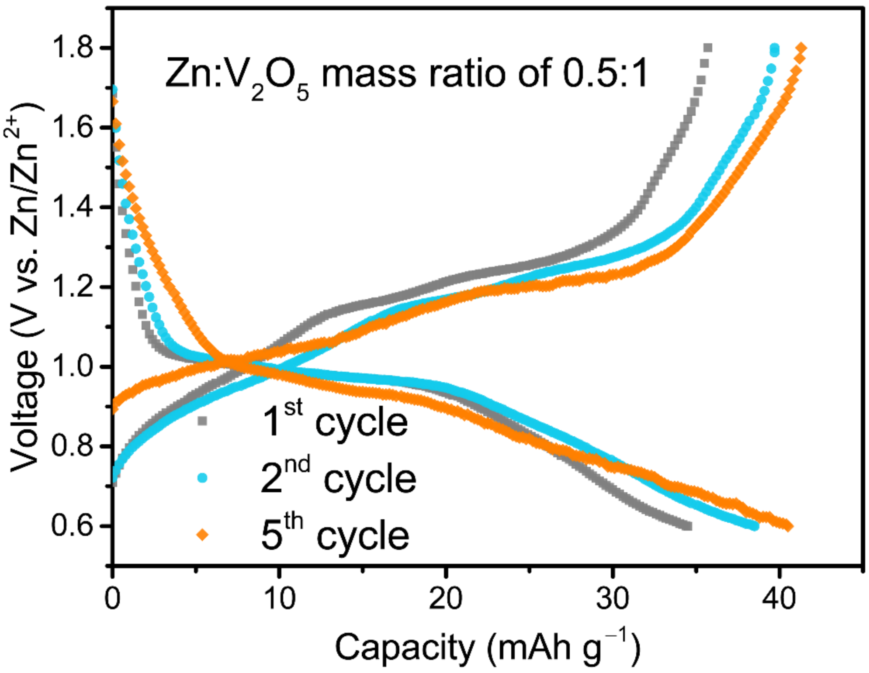

Supplementary Fig. 43 The charge/discharge curves of Zn/V_2O_5 cell with ZES at 2.81 mA cm^{-2} (Zn:V_2O_5 mass ratio of 0.5:1, after activation)**.**

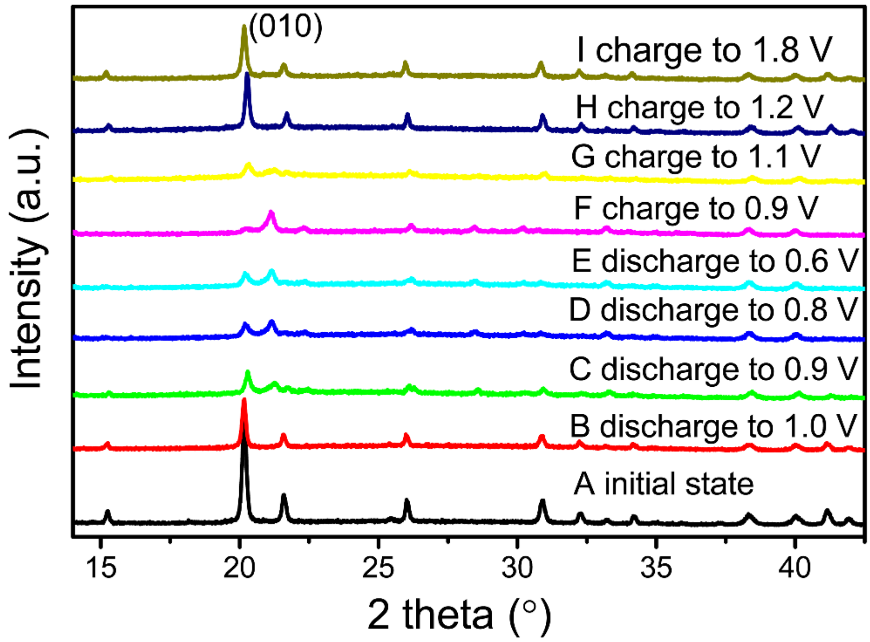

Supplementary Fig. 44 XRD patterns of the V_2O_5 cathode at different voltage states during the first charge/discharge cycle in ZES.

Supplementary Tables

Supplementary Table 1. MD simulation details of ZESs

Electrolyte molar ratio ($Zn(TFSI)_2$/Ace)	1:4	1:7
Number of $Zn(TFSI)_2$ per box	100	100
Number of Ace per box	400	700
Total number of atoms	6700	9400
Simulation box size (Å^3)	52.3×52.3×52.3	55.7×55.7×55.7
MD, density (kg m^{-3})	1000	1000
Bias temperature (K)	298	298

Supplementary Table 2.The lowest-eutectic temperatures of ZESs with different molar ratios (based on Supplementary Fig. 2).

Symbol	Electrolyte molar ratio ($Zn(TFSI)_2$/Ace)	Lowest-eutectic temperature (°C)
A	1:9	−51.51
B	1:7	−51.32
C	1:5	−36.20
D	1:4	−35.70

Supplementary Table 3. Summary of the viscosities, ionic conductivities and activation energies for each composition (obtained on the basis of Supplementary Fig. 13).

Symbol	Molar ratio ($Zn(TFSI)_2$/Ace)	E_a (kJ mol^{-1})	A	σ (mS cm^{-1}) 25 °C	Viscosity (Pa·s)
A	1:9	41.54	9189	0.51	0.533
B	1:7	45.49	28324	0.31	0.789
C	1:5	48.34	55111	0.19	1.89
D	1:4	46.82	27097	0.16	2.13

Supplementary Table 4. CE of Zn plating/stripping of the reported concentrated Zn aqueous electrolytes.

	Working electrode	Counter electrode	CE	Ref
30 m $ZnCl_2$	Zn	Zn	95%	[3]
$ZnCl_2 \cdot 2.3H_2O$	Mo	Zn	98.7%	[4]
2 M $ZnSO_4$	Pt	Zn	75%	[5]
2 M $Zn(CH_3COO)_2$	Pt	Zn	80%	[5]

Supplementary Table 5. FTIR band assignment for the ZES and the SEI layer (based on Supplementary Fig. 30). Vibration cm^{-1}.

Ace	$Zn(TFSI)_2$	ZES	SEI	Band assignment	Ref
1665		1663	1664	υ (C=O)	[6]
1394			1402	υ (CN)	[6]
	1350	1350	1354	υ_{as} (SO_2)	[7]
	1201	1197	1203	υ_{as} (CF_3)	[7]
	1143	1144	1139	υ_s (SO_2)	[7]

Supplementary Notes

Supplementary Note 1: Solution structure analysis of ZESs.

Ace is a simple and typical donor molecule for deep eutectic solvents (DESs) due to its dipolar nature and has a strong ability to coordinate or H-bond with metal cations and anions by its two polar groups (C=O group and NH_2 group)[8-11]. The molten Ace also provides a high dielectric permittivity of 60 at 80 °C[6]. Its acid-base properties are similar to those of water, and a variety of organic and inorganic compounds have been found to be soluble in Ace[6]. It is known that the physical/electrochemical properties of DESs are adaptable and dependent upon the donor moiety. DESs will be endowed with the advantages of relatively low viscosity and high ionic conductivity when Ace serves as the HBD[12], which has also been demonstrated by our previous works[11,13]. Ace-based DESs have been reported as an alternative system for developing Zn electrolytes[14]. Additionally, amides are generally low-cost, chemically stable and environmentally friendly, which is beneficial for large-scale practical applications[15].

FTIR spectra of the ZESs are distinct from those of pure $Zn(TFSI)_2$ and Ace (shown in Supplementary Fig. 5). With the gradual addition of $Zn(TFSI)_2$ into Ace, the broad peak centered at 3351 cm^{-1} moves to 3369 cm^{-1} with an additional peak appearing at 3458 cm^{-1}. The latter corresponds to the free N–H stretch, while the former may be originated from the interaction between the NH_2 group of Ace and the SO_2 group of $TFSI^-$ via hydrogen bonding[7,9]. More obvious change appears at 1665 cm^{-1}, which is assigned to the C=O stretching mode of Ace. It red shifts slightly to 1654 cm^{-1} and the peak width increases in ZESs compared with in pristine Ace. This is in line with the formation of metal-oxygen bond between Zn^{2+} and the C=O group[6,9,16].

Supplementary Note 2: Theoretical simulations of solution structure in ZESs.

The DFT geometry optimization of the $[ZnTFSI(Ace)_4]^+$ complex started from a configuration with four Ace molecules bound to a Zn^{2+} cation, but the optimized geometry instead converging to a configuration with only three of the Ace molecules bound to the Zn^{2+} cation while the other Ace is directed away from the central cation (Supplementary Fig. 9f). Moreover, the DFT geometry optimization of the $[ZnTFSI_2(Ace)_4]$ complex started from a configuration with four Ace molecules bound to a Zn^{2+} cation, but the optimized geometry is similar to the $[ZnTFSI(Ace)_4]^+$ case (Supplementary Fig. 10h).

The DFT binding energies shown in Supplementary Fig. 11 indicate that the magnitude of the complex stability follows the order $E\,(C_1\text{-O-}\Pi) < E\,(C_1\text{-O-I}, C_1\text{-N-I}) < E\,(C_2\text{-O-}\Pi)$ with the $E\,(C_2\text{-O-}\Pi)$ being the most stable. Furthermore, these date also verify the tendency of the C=O group of Ace and the two O atoms of $TFSI^-$ (two O atoms are located on different S atoms) to coordinate with the central Zn^{2+} ion (bidentate coordination).

Supplementary Note 3: Determination of the control electrolyte group.

In order to ensure the consistency between samples in terms of anion and cation, $Zn(TFSI)_2$ is also applied in the control electrolyte group. However, due to the high charge density of Zn^{2+}, $Zn(TFSI)_2$ is difficult to dissociate in organic solvents. Thus, water is used as the solvent since it can make $Zn(TFSI)_2$ more easily dissociated, though the Zn salt will be precipitated in the 2 M $Zn(TFSI)_2$ after resting for 24 hours, as shown in Supplementary Fig. 14a. Here, taking into account the pH, the ionic conductivity as well as the electrochemical performance (Supplementary Fig. 15) of different concentrations, we choose 1 M $Zn(TFSI)_2$ as the control sample (Supplementary Fig. 14b).

Supplementary Note 4: Electrochemical stability of various electrolytes.

It is evident that due to the O_2 evolution reaction, the potential window of 1 M $Zn(TFSI)_2$ is restricted to 1.9 V (*vs.* Zn/Zn^{2+}) and yellowing of the separator caused by corrosion of SS was observed after testing (Supplementary Fig. 16a, inset I). As a contrast, the ZES shows a much higher anodic stability (2.4 V *vs.* Zn/Zn^{2+}) without yellowing of the separator (Supplementary Fig. 16a, inset Π lower). When the voltage is >2 V, the O_2-evolution reaction of 1 M $Zn(TFSI)_2$ increased the acidity of solution, thereby further exacerbating the corrosion of SS and Zn electrode. However, the surface of Zn electrode in ZES remains smooth without corrosion (Supplementary Fig. 16a, inset Π). Before the preparation of ZESs, the raw materials (Ace and $Zn(TFSI)_2$) were dried to remove residual water. The oxidation current of ZES is decoupled from the O_2-evolution reaction, but attributed to the decomposition of electrolyte components. In addition, the working electrode for testing the electrolyte electrochemical window has an impact on the oxidation reactions (Supplementary Fig. 16b)[17].

Supplementary Note 5: Electrochemical performance of the Zn/V_2O_5 cells in different electrolytes.

Remarkably, the Zn/V_2O_5 cell with ZES displays a maximum discharge capacity of 186 mA h g^{-1} at 100 mA g^{-1} and a high remaining discharge capacity of 110 mA h g^{-1} at the 200th cycle (Supplementary Fig. 39b). The better performance of the Zn/V_2O_5 cell with ZES can be mainly attributed to the stability of the Zn anode protected by SEI layer. In contrast, the specific capacity decreased by ~50% after the first 60 cycles when 1 M Zn(TFSI)$_2$ is used as electrolyte.

Supplementary Methods

Chemicals: Zinc bis(trifluoromethanesulfonyl)imide ($Zn(TFSI)_2$, 99%) and acetamide (Ace) (99%) purchased from Solvionic and Aladdin, respectively, were low-pressure dried at 65 °C for 12 h prior to use. Zinc acetate ($Zn(CH_3COO)_2$), $Zn(BF_4)_2$ and $Zn(ClO_4)_2$ were purchased from Sigma-Aldrich. The viscosity of the electrolytes was measured by a rotational rheometer (MCR301, Anton Paar Instrument).

Electrochemical Measurements: Cyclic voltammetry (CV) was carried on an electrochemical workstation (VMP-300, Bio-Logic Science Instruments Co). The electrochemical stability windows of the electrolytes were measured on the inert current collector (stainless steel (SS) foil or Ti foil). Electrochemical impedance spectroscopy (EIS) data were acquired using the electrochemical workstation with a frequency range from 7 MHz to 0.1 Hz. Galvanostatic charge-discharge measurements were performed with a LAND CT2001A Battery Cycler (Wuhan, China).

Ionic conductivities were tested by two blocking electrodes (SS) and calculated according to the following equation [1]:

$$\sigma = \frac{l}{R_{\mathrm{b}} \cdot S} \quad [1]$$

where R_b represents the resistance according to EIS measurement, l represents the thickness of the membrane, and S is the area of the contact between SS and electrolyte.

The variation in ionic conductivity as a function of temperature of ZESs (shown in Supplementary Fig. 13a) follows the classical Arrhenius type of behavior as equation [2]

$$\ln\sigma = \ln\mathrm{A} - E_{\mathrm{a}} / RT \quad [2]$$

where σ is the conductivity of the molten solvent in mS cm^{-1}, A is the pre-exponential factor, E_a is the activation energy expressed in kJ mol^{-1}, R is the gas constant in J K^{-1} mol^{-1}, and T is the temperature in kelvin.

To get the detailed ionic conductivity of the SEI layer (based on Supplementary Fig. 31), the following equation was used [3][18-20]:

$$\sigma = 2\pi f_{\max}\varepsilon_0\varepsilon_r \qquad [3]$$

where σ represents the ionic conductivity of the in-situ-formed SEI skeleton, and ε_0 and ε_r are the permittivities of free space and the SEI layer ($\varepsilon_0 = 8.9 \times 10^{-12}$ F/m and $\varepsilon_r = 10.0$ F/m based on previous reports[18-20]), respectively.

Moreover, the test method for the activation energy for Zn^{2+} diffusion through SEI layer in Supplementary Fig. 32 is mainly according to Xu et al. and Zhang et al.'s reports[21,22].

Zn^{2+} transference number was evaluated in symmetrical Zn battery combined by EIS before and after the chronoamperometry (CA) test (shown in Supplementary Fig. 18a), and calculated by the following equation [4]:

$$T = \frac{I_S(\Delta V - I_0 R_0)}{I_0(\Delta V - I_S R_S)} \qquad [4]$$

where ΔV is the voltage polarization applied, I_s and R_s are the steady state current and resistance, respectively, and I_0 and R_0 are the initial current and resistance, respectively. The applied voltage polarization here is 5 mV.

A vital important parameter when evaluating electrolyte systems for electrochemical applications is the diffusivity of the electroactive species through the electrolyte, which can limit the charge/discharge rate capability of devices[23]. Chronoamperometry experiments can be performed to determine the diffusion coefficient of the reactant Zn species in a diffusion-limited process via the Cottrell Equation [5]:

$$i = \frac{nFACD^{\frac{1}{2}}}{\pi^{\frac{1}{2}}t^{\frac{1}{2}}} \qquad [5]$$

In this equation, i is the current (A), n is the number of the transferred electrons (2), F is Faraday's Constant (96,485 C mol^{-1}), C is the concentration of the electroactive species, A is the active surface area of the electrode-electrolyte interface, D is the

diffusion coefficient ($cm^2\ s^{-1}$), and t is time (s). The diffusion coefficient can be deduced from the slope of a plot of i vs. $t^{-1/2}$. From the graph in Supplementary Fig. 18b, the diffusion coefficient of the active Zn species in ZES electrolyte was found to be $1.66\times10^{-6}\ cm^2\ s^{-1}$. Compared to electrolyte systems investigated by Simons et al. and Xu et al.[23-24], this value appears to be significantly higher.

The peak current (i) and the sweep rate (υ) (based on CV, shown in Supplementary Fig. 38) abide by the relationship [6]:

$$i = a\upsilon^b \qquad [6]$$

where both a and b are adjustable parameters. Meanwhile, the b value can be obtained by the slope of the log (i) vs. log (υ) plot. When the electrochemical behavior is dominated by the ionic diffusion process, the b value is close to 0.5.

Theoretical simulations: Molecular dynamic (MD) simulations were performed on the electrolyte mixtures ($Zn(TFSI)_2$ and Ace) with various composition ratios to observe the structure changes of them. First, the optimized electrolyte molecules were packed in a periodic box to construct the bulk systems, the compositions of simulated electrolytes are given in Supplementary Table 1. The molar ratios of $Zn(TFSI)_2$/Ace used in our simulations were 1:4 and 1:7. The simulation cells contained 100 $Zn(TFSI)_2$ and 400 Ace and 100 $Zn(TFSI)_2$ and 700 Ace, respectively. Subsequently, all mixture systems were equilibrated by NPT (*i.e.*, isothermalisobaric) MD simulations for 2ns at 353 K and atmospheric pressure, followed by NPT (*i.e.*, isothermal) MD simulations for 10 ns with a 1 fs time step. All MD simulations were performed using the Forcite code with Universal force field[25]. The temperature was controlled by a Nose-Hoover Langevin (NHL) thermostat and the pressure was controlled by a Berendsen barostat[26,27]. The Ewald scheme[28,29] and atom-based cutoff method (*i.e.*, a radius of 15.5 Å) were applied to treat electrostatic and van der Waals (vdW) interactions, respectively. All the partial atomic charges were defined using the Universal force field.

Supplementary References

1. Herstedt, M. *et al.* Spectroscopic characterization of the conformational states of the bis (trifluoromethanesulfonyl)imide anion ($TFSI^-$). *J. Raman Spectrosc.* **36**, 762-770 (2005).

2. Dilasari, B., Jung, Y. & Kwon, K. Effect of water on the stability of zinc in 1-butyl-1-methylpyrrolidinium bis(trifluoromethylsulfonyl)imide ionic liquid. *J. Ind. Eng. Chem.* **45**, 375-379 (2017).

3. Zhang, C. *et al.* A $ZnCl_2$ water-in-salt electrolyte for a reversible Zn metal anode. *Chem.Commun.* **54**, 14097-14099 (2018).

4. Chen, C. Y., Matsumoto, K., Kubota, K., Hagiwara, R. & Xu, Q. A Room-Temperature Molten Hydrate Electrolyte for Rechargeable Zinc-Air Batteries. *Adv. Energy Mater.* **9**, 1900196 (2019).

5. Wang, F. *et al.* Highly reversible zinc metal anode for aqueous batteries. *Nat. Mater.* **17**, 543, (2018).

6. Narayanan, N. S. V., Ashokraj, B. V. & Sampath, S. Physicochemical, electrochemical, and spectroscopic characterization of Zinc-based room-temperature molten electrolytes and their application in rechargeable batteries. *J. Electrochem. Soc.* **156**, A863-A872 (2009).

7. Rey, I., Lassegues, J. C., Grondin, J. & Servant, L. Infrared and Raman study of the PEO-LiTFSI polymer electrolyte. *Electrochim. Acta.* **43**, 1505-1510 (1998).

8. Liang, H. Y. *et al.* New binary room-temperature molten salt electrolyte based on urea and LiTFSI. *J. Phys. Chem. B* **105**, 9966-9969 (2001).

9. Hu, Y. S., Wang, Z. X., Li, H., Huang, X. J. & Chen, L. Q. Spectroscopic studies on the cation-anion, cation-solvent and anion-solvent interactions in the $LiCF_3SO_3$/acetamide complex system. *Spectrochim. Acta. A* **61**, 403-411 (2005).

10. Smith, E. L., Abbott, A. P. & Ryder, K. S. Deep eutectic solvents (DESs) and their applications. *Chem. Rev.* **114**, 11060-11082 (2014).

11. Zhang, J. *et al.* Amide-based molten electrolyte with hybrid active ions for rechargeable Zn batteries. *Electrochim. Acta* **280**, 108-113 (2018).

12. Zhang, Q., Vigier, K. D. O., Royer, S. & Jerome, F. Deep eutectic solvents: syntheses, properties and applications. *Chem. Soc. Rev.* **41**, 7108-7146 (2012).

13. Zhao, J. *et al.* "Water-in-deep eutectic solvent" electrolytes enable zinc metal anodes for rechargeable aqueous batteries. *Nano Energy* **57**, 625-634 (2019).

14. Mainar, A. R. *et al.* An overview of progress in electrolytes for secondary zinc-air batteries and other storage systems based on zinc. *J. Energy Storage* **15**, 304-328 (2018).

15. Venkata Narayanan, N. S., Ashokraj, B. V. & Sampath, S. Ambient temperature, zinc ion-conducting, binary molten electrolyte based on acetamide and zinc perchlorate: Application in rechargeable zinc batteries. *J. Colloid Interf. Sci.* **342**, 505-512 (2010).

16. Yu, Z. X. *et al.* A series of LiI/acetamide phase transition electrolytes and their applications in dye-sensitized solar cells. *Electrochim. Acta.* **55**, 895-902 (2010).

17. Zhang, Z. *et al.* Novel Design Concepts of Efficient Mg-Ion Electrolytes toward High-Performance Magnesium-Selenium and Magnesium-Sulfur Batteries. *Adv. Energy Mater.* **7**, 1602055 (2017).

18. Bouchet, R., Lascaud, S. & Rosso, M. An EIS study of the anode Li/PEO-LiTFSI of a Li polymer battery. *J. Electrochem. Soc.* **150**, A1385-A1389 (2003).

19. Liu, Y. *et al.* An artificial solid electrolyte interphase with high Li-ion conductivity, mechanical strength, and flexibility for stable lithium metal anodes. *Adv. Mater.* **29**, 1605531 (2017).

20. Bruce, P. & Krok, F. Studies of the interface between V_6O_{13} and poly (ethylene oxide) based

electrolytes. *Electrochim. Acta*. **33**, 1669-1674 (1988).
21. Xu, K., Lam, Y., Zhang, S. S., Jow, T. R. & Curtis, T. B. Solvation sheath of Li^+ in nonaqueous electrolytes and its implication of graphite/electrolyte interface chemistry. *J. Phys. Chem. C* **111**, 7411-7421 (2007).
22. Zhang, X. Q. *et al.* Highly stable lithium metal batteries enabled by regulating the solvation of lithium Ions in nonaqueous electrolytes. *Angew. Chem. Int. Ed.* **57**, 5301-5305 (2018).
23. Simons, T. J., MacFarlane, D. R., Forsyth, M. & Howlett, P. C. Zn electrochemistry in 1-ethyl-3-methylimidazolium and N-butyl-N-methylpyrrolidinium dicyanamides: promising new rechargeable Zn battery electrolytes. *Chemelectrochem* **1**, 1688-1697 (2014).
24. Xu, M., Ivey, D. G., Qu, W. & Xie, Z. Improved Zn/Zn(II) redox kinetics, reversibility and cyclability in 1-ethyl-3-methylimmidazolium dicyanamide with water and dimethyl sulfoxide added. *J. Power Sources* **252**, 327-332 (2014).
25. Sun H. COMPASS: an ab initio force-field optimized for condensed-phase applications overviewwith details on alkane and benzene compounds. *J. Phys. Chem. B* **102**, 7338-7364 (1998).
26. Samoletov, A. A., Dettmann, C. P., & Chaplain, M. A.. Thermostats for “slow” configurational modes. *J. Stat. Phys.* **128**, 1321-1336 (2007).
27. Berendsen, H. J., Postma, J. V., van Gunsteren, W. F., DiNola, A. R. H. J., & Haak, J. R. Molecular dynamics with coupling to an external bath. *J. Chem. Phys.* **81**, 3684-3690 (1984).
28. Ewald P. P. Die Berechnung optischer und elektrostatischer Gitterpotentiale. Ann. Phys. **369**, 253-287 (1921).
29. Tosi M. P. Cohesion of ionic solids in the Born model. *Solid State Phys.* **16**, 1-120 (1964).
